# Know Thy Selves: Learning to Understand Oneself Increases the Ability to Understand Others

**DOI:** 10.1007/s41465-017-0023-6

**Published:** 2017-05-16

**Authors:** Anne Böckler, Lukas Herrmann, Fynn-Mathis Trautwein, Tom Holmes, Tania Singer

**Affiliations:** 1 Department of Social Neuroscience, Max Planck Institute for Human Cognitive and Brain Science, Stephanstraße 1, 04103 Leipzig, Germany; 2 University of Würzburg, Würzburg, Germany; 3 Western Michigan University, Kalamazoo, MI USA

**Keywords:** Internal Family System, Theory of Mind, Self, Inner parts, Contemplative mental training

## Abstract

Understanding others’ feelings, intentions, and beliefs is a crucial social skill both for our personal lives and for meeting the challenges of a globalized world. Recent evidence suggests that the ability to represent and infer others’ mental states (Theory of Mind, ToM) can be enhanced by mental training in healthy adults. The present study investigated the role of training-induced understanding of oneself for the enhanced understanding of others. In a large-scale longitudinal study, two independent participant samples (*N* = 80 and *N* = 81) received a 3-month contemplative training. This training focused on perspective taking and was inspired by the Internal Family Systems model that conceives the self as being composed of a complex system of inner personality aspects. Specifically, participants practiced perspective taking on their own inner states by learning to identify and classify different inner personality parts. Results revealed that the degree to which participants improved their understanding of themselves—reflected in the number of different inner parts they could identify—predicted their improvements in high-level ToM performance over training. Especially the number of identified parts that were negatively valenced showed a strong relation with enhanced ToM capacities. This finding suggests a close link between getting better in understanding oneself and improvement in social intelligence.

**Electronic supplementary material:**

The online version of this article (doi:10.1007/s41465-017-0023-6) contains supplementary material, which is available to authorized users.

## Introduction

As citizens of the twenty-first century, we face many problems of an industrialized and globalized world. Tensions between countries, cultures, and religions are rising; wars and political instabilities drive millions of people to leave their homes and seek refuge; and the consequences of climate change become ever more visible. Skillful solutions to these problems will require cooperation across nationalities and cultures. In order to achieve this goal, we need to put ourselves in the shoes of others—considering the viewpoint of those who hold different political and religious views and who are seeking shelter in our countries as well as bearing in mind the needs of future generations. Taking the cognitive perspective of other people is relevant for peaceful and successful interactions not only on an inter-cultural scale, but also in families, schools, and workplaces, both when communicating with acquaintances and with complete strangers. The importance of our ability to perceive other people as thinking and feeling beings has been emphasized in developmental and clinical domains: Impaired interaction and communication skills as well as aggressive and abusive behaviors towards others, for instance, have repeatedly been linked to reduced capacities or inclinations to take others’ perspectives into account (e.g., Fonagy 2003; Frith and Happe 1994). Also in healthy adult populations, research suggests that taking others’ perspectives enhances cooperation (Paal and Bereczkei 2006), reduces stereotyping (Galinsky and Moskowitz 2000; Todd et al. 2012), and widens our “circle of moral regard” to the inclusion of formerly stigmatized groups (Batson et al. 1997). But how can we enhance this capacity? And, more specifically, does the enhancement of a better understanding of the self help better understanding others?

The socio-cognitive ability to take other people’s perspective, to infer and reason about their mental states such as thoughts, beliefs, and intentions, is referred to as Theory of Mind (ToM), mentalizing or cognitive perspective taking (Frith and Frith 2003; Premack and Woodruff 1978; Singer 2012). Over the last decades, neuroscientific research has revealed that processing others’ mental states leads to enhanced activation in a network including temporo-parietal junction (TPJ), superior temporal sulcus, temporal poles, and medial prefrontal cortex (mPFC) (Bzdok et al. 2012; Dodell-Feder et al. 2011; Kanske et al. 2015; Schurz et al. 2014). This cognitive route to understanding others has been distinguished from an affective route to understanding others (i.e., empathic sharing of another’s bodily or emotional state; Vignemont and Singer 2006) both on the level of brain and behavior (Kanske et al. 2015; Kanske et al. 2016; Lamm et al. 2011; Singer 2006). A main focus of research on ToM has been the questions whether and to what degree the ability to represent conspecifics’ mental states is also present in our closest biological relatives—chimpanzees (Premack and Woodruff 1978), at what age children develop this capacity (Wellman and Estes 1986), and how this capacity is impaired in psychopathologies such as autism (Baron-Cohen et al. 1985; Bird et al. 2010; Frith and Happe 1994; Hoffmann et al. 2015; for a review, see Singer 2012).

In addition, previous studies have shown that cognitive perspective taking trainings that entail explicit familiarization with the concept of mental states and the practice of mentalizing tasks can promote the development of ToM capacity in children (Ding et al. 2015; Lecce et al. 2014a, b) and slow down the decline of ToM performance in the elderly (Cavallini et al. 2015; Henry et al. 2013). Initial evidence suggests that even in healthy adults, meditation-based trainings can improve the understanding of emotional expressions (assessed by the Reading the Mind in the Eyes test; Mascaro et al. 2013; Schurz et al. 2014). Crucially, recent evidence from the *ReSource Project*, a large-scale longitudinal training study (Singer et al. 2016), showed reliable increase in high-level ToM performance in healthy adult populations after 3 months of mental training in metacognition and perspective taking (Trautwein et al. under review) in an ecologically valid task (EmpaToM, see Kanske et al. 2015).

However, the underlying mechanisms for these promising changes in ToM performance are still poorly understood. In the present study, we therefore investigated how practicing the ability to understand one’s own mental states, which was part of the above-mentioned perspective training, relates to the observed improvements in ToM. Common sense and established sayings clearly advocate that “you have to know yourself to know others.” More generally, decades of social neuroscience research in the domain of empathy, action understanding, and mentalizing suggest that understanding others’ feelings, actions, or even mental states rely on activating representations necessary to understand your own feelings (Singer 2012), actions (Keysers and Gazzola 2009), or mental states (Mitchell 2009). Neuroimaging studies show, for instance, that partly similar brain areas are involved in reasoning about oneself (e.g., on one’s personality traits) and reasoning about others (e.g., mirror neuron systems, Gallese and Goldman 1998; Goldman 2006; mPFC, TPJ, Lombardo et al. 2010; mPFC, Mitchell and Phillips 2015). Similarly, in a review on theoretical models and empirical data from neuroimaging and clinical studies, Dimaggio et al. (2008) concluded that the capacities for understanding oneself and others are “semi-independent skills” which may enhance each other. So far, however, within-subject data on the link between learning to take the perspective on oneself and taking the cognitive perspective of others is lacking, precluding any form of mechanistic interpretation of existing neuroimaging findings of shared brain mechanisms involved in mentalizing about self and others.

**Fig. 1:**
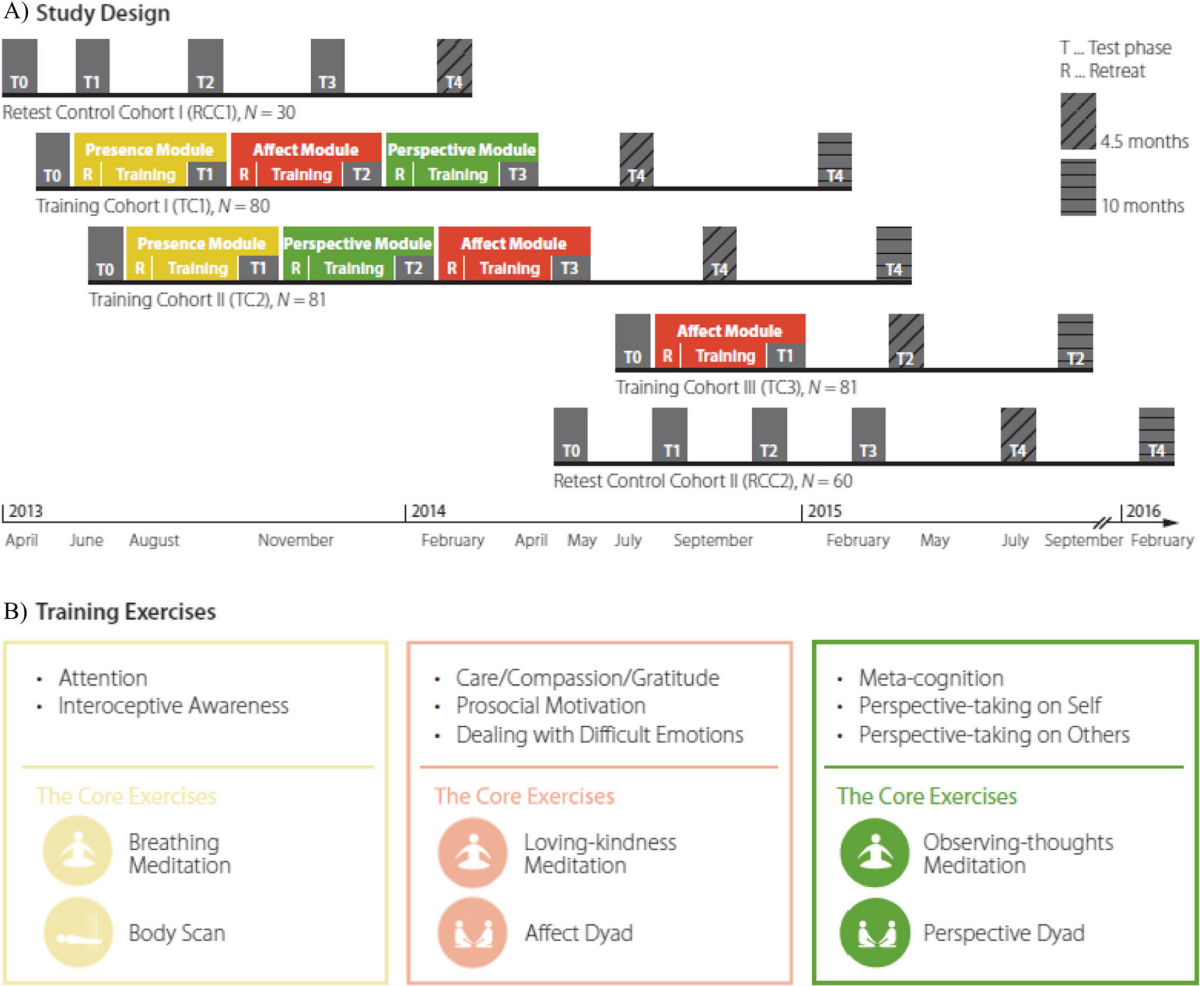
Design and training exercises of the ReSource study. **a** Timeline of training modules (*colored areas*) and data collection phases (*gray areas*, T0–T4) for the three training cohorts (TC1, TC2, TC3) and the retest control cohorts (RCC1 and RCC2). TC1 and TC2 completed all three modules and differed only in the order of the Affect and Perspective module. R in colored boxes indicates retreats. RCC1 and RCC2 were split for logistical reasons into two smaller cohorts but are jointly analyzed. Both retest control cohorts completed all measurements but did not receive any training. **b** Illustration of the core exercises of the three modules (*left to right*): Presence (*yellow*), Affect (*red*), Perspective (*green*)

The question whether learning to understand oneself is related to improvements in ToM performance could be addressed in the context of the aforementioned training study, the *ReSource Project* (Singer et al. 2016). In this project, participants completed one or all of three distinct mental training modules (each lasting for 3 months), focusing on the cultivation of attention and interoceptive awareness (“Presence Module”), of socio-affective qualities such as care, compassion, gratitude, and prosocial motivation (“Affect Module”), or of socio-cognitive abilities such as metacognition and perspective taking on self and others (“Perspective Module”). Another participants sample served as retest controls and did not partake in any training (see Fig. 1, panel a for an overview of the study design). The present study focuses on the Perspective Module because this module selectively induced significant increases in ToM performance (Trautwein et al. under review).

During the Perspective Module, participants practiced two core exercises (see Fig. 1, panel b). The first was an Observing-thoughts meditation, a contemplative method aiming to foster metacognitive awareness of and detachment from one’s thinking process (Ricard 2010). The second core exercise was a contemplative dyad (“perspective dyad,” Kok and Singer 2017) that aimed to enhance the capacity of cognitive perspective taking on one’s own and others’ mental processes. This contemplative dyadic practice is inspired by previous research in ToM (for review see Singer 2012) and the Internal Family Systems (IFS) model (Holmes 2011; Schwartz 2013; for promising results of a proof-of-concept study, see Shadick et al. 2013). The IFS model regards a person’s personality as composed of relatively discrete subpersonalities—inner parts—each of which possesses its own characteristic set of cognitions, affects, and behaviors (Schwartz 1995). In the context of the *ReSource Project*, the IFS model was extracted from its typical therapeutic context and implemented to allow participants to identify and get to know different inner parts (as forms of their own emotional, cognitive, or behavioral patterns) (Kok and Singer 2017; Singer et al. 2016). Participants were taught to identify their own inner parts, explored how being identified with different inner parts affects their everyday experiences, and learned to recognize other people’s inner parts (for details, see method section).

Because such a contemplative approach to describing and cultivating one’s own mental patterns by means of inner parts is novel in psychological research, our first goal was to describe which types of inner parts participants came up with and to validate this new approach by investigating how the inner parts participants identified throughout the 3-month Perspective Module related to established psychometric trait affect measures. Since affective valence is one of the most defining features of the inner parts (Holmes 2011; Schwartz 1995), the key question was whether the valence of participants’ parts reflected their trait affect assessed by trait questionnaires. Specifically, we investigated whether participants who identified more positive inner parts and who showed a more positive average valence of inner parts also reported more positive trait affect, and vice versa for negative parts and affect.

By engaging in inner parts work in combination with the daily perspective dyad, participants effectively practiced taking the perspective of their own and others’ inner parts. The underlying assumption is that familiarizing oneself with one’s own inner part system will be reflected in discerning a greater number and variety of different inner parts over time (Schwartz 1995). Especially identifying negative parts seems the more demanding piece of the process, requiring to overcome an initial resistance against identifying with negatively valenced memories and emotions (Holmes 2011). The second and main goal of the present study, hence, was to investigate the link between the number of identified negative and positive parts throughout the Perspective Module (as an indicator of performance in perspective taking on self) and the increase in ToM performance assessed before and after the 3-month perspective training (as an indicator for the capacity to take the perspective on others). Specifically, if a better understanding of oneself does indeed relate to a better understanding of others’ mental states, identifying a larger number of inner parts in the course of the training should be accompanied by greater improvements in ToM performance subsequent to the training. Especially the identification of negatively valenced inner parts was expected to be related to a training-related increase in ToM.

## Methods

### Participants

In total, 332 participants (197 female; mean age = 40.74, SD = 9.24; age range = 20–55) took part in the ReSource study (for details of recruitment and assignment, see also Singer et al. 2016) and were randomly assigned to three training cohorts, TC1, TC2, and TC3 (*N*s = 80/81/81), and a retest control cohort (RCC; *N* = 90). As illustrated in Fig. 1, TC1 and TC2 underwent three distinct training modules (Presence Module, Affect Module, Perspective Module) in different orders, TC3 completed the Affect Module only, and RCC was tested at similar time points as the training cohorts, but without undergoing any form of mental training (for details, see Singer et al. 2016). In the present study, only data from TC1 and TC2 were included because only participants of these cohorts completed the Perspective Module. Thirteen participants dropped out before undergoing this module, data of seven participants was lost due to technical data-storage difficulties with the custom-made platform. The remaining sample entailed data of 141 individuals (81 female, mean age = 40.94, SD = 9.39).

Participants were volunteers recruited from the Berlin and Leipzig area (Germany) via various means, for example, flyers, local newspaper, and TV and radio announcements between winter 2012/2013 and winter 2013/2014. All participants underwent an extensive screening procedure to ensure mental health (e.g., Major Depression Inventory, Bech et al. 2001; DIA-X for axis I disorders for DSM-IV, Wittchen and Pfister 1997; Structured Clinical Interview for DSM-IV, SKID-I, Wittchen et al. 1997), abstinence from drugs and medication use, and naivety concerning contemplative practices and psychological measures. For more details about selection criteria, see Singer et al. (2016). All participants signed informed consent prior to participation. The study was approved by the Research Ethics Committee of the University of Leipzig, number 376/12-ff and the Research Ethics Committee of the Humboldt University in Berlin, numbers 2013-02, 2013-29, and 2014-10. The study was registered with the Protocol Registration System of ClinicalTrials.gov under the title “Plasticity of the Compassionate Brain” (Identifier: NCT01833104).

### ReSource Project Design and Model

As illustrated in Fig. 1, participants underwent a 9-month mental training program composed of three 3-month modules (Presence, Affect, and Perspective Module). Each module focused on cultivating distinct mental and affective capacities, such as present-moment and interoceptive awareness and attention (Presence Module), loving kindness, gratitude and compassion, dealing with difficult emotions, and prosocial motivation (Affect Module), and metacognitive skills and perspective taking on self and others (Perspective Module). Participants practiced a set of meditation exercises, which have been commonly used for the cultivation of the respective abilities in contemplative traditions and in Western clinical psychology (Holmes 2011; Kabat-Zinn 2003; Neff 2011; Ricard 2008). The meditation exercises combined both traditional meditation-based as well as more novel dyadic contemplative practices (Kok and Singer 2017; Noyes 1998).

Each training module lasted 13 weeks of which the first 8 weeks were used to teaching and developing the respective contemplative practices. During the final 5 weeks, the respective abilities (e.g., ToM) were assessed while participants continued to deepen their practice (Singer et al. 2016). Qualified meditation teachers with ample experience in contemplative traditions were specifically recruited for the study and taught all training modules. At the beginning of each training module, participants attended a 3-day retreat under the supervision of the meditation teachers who introduced participants to the core exercises of the respective trainings. These core exercises were then individually practiced on a daily basis for 30 min at home. In addition, participants attended a weekly session with the meditation teachers. As noted above, for the present paper, only data from the Perspective Module are of interest.

### The Perspective Module: Inner Parts Work and Dyadic Practice

The Perspective Module intended to develop metacognitive skills, detachment from habitual cognitive processes as well as perspective taking on self and others. The Perspective Module began with a 3-day retreat in which participants were familiarized with the module’s core exercises and continued with a 3-month practicing period. During this period, participants attended 2-h weekly sessions in groups of 20 participants led by two contemplative practice trainers. The core practices included an Observing-thoughts meditation and a dyadic practice, the so-called perspective dyad. During the Observing-thoughts meditation, participants were seated with their eyes closed and paid special attention to their thinking. Labels were used to classify the content of the thoughts along the dimensions of me/other, past/future, or positive/negative. The objective was to observe thoughts as mental events or natural phenomena rather than taking them for accurate depictions of reality, to observe thoughts without getting involved in them, and to de-identify from thoughts, thereby gaining insight into the workings of the mind and greater flexibility with regard to successive thoughts, feelings, and behaviors. The daily contemplative dyad was based on the identification of inner parts, influenced by the Internal Family Systems model (IFS), a therapeutic approach developed by Schwartz (1995) and further outlined by Holmes (2011). Our main focus is on the dyadic practice which focuses on cultivating perspective taking on self and others and which we will outline in the following sections.

#### Inner Parts Work

Participants trained perspective taking on the self through the concept of inner parts (Holmes 2011; Schwartz 1995). Inner parts according to IFS are relatively discrete subpersonalities which are each characterized by specific affective, cognitive, and behavioral patterns (Schwartz 1995). The IFS model loosely classifies inner parts based on their therapeutic relevance into the categories of *Managers*, *Exiles*, and *Firefighters* (Schwartz 1995). *Managers*, for instance, intend to adapt the person to the demands of the external world and are often reflected in behavioral and cognitive patterns aiming at rationally structuring the persons’ everyday life. *Exiles* are parts which are burdened with severe, negative affect due to past traumatizing experiences (Schwartz 1995). Both *Managers* and *Firefighters* attempt to keep *Exiles* from entering conscious awareness, e.g., by distracting the person from the negative or threatening content by overeating or excessive overworking. Beyond this psychotherapeutic application, Holmes (2011) suggested various types of inner parts relevant for a non-clinical population, such as *Pleasure Parts* which are driven by the pleasure principle of immediate satisfaction of physical needs or *Caring Parts* which exhibit feelings of empathy and closeness and a caring motivation. In an initial reflection phase that took part during the 3-day retreat in the beginning of the Perspective Module and that is typically employed in the introduction of the IFS model, participants were asked to identify the inner parts that would be dominant in exemplary situations, such as playing with a child or giving an important talk. Each participant registered the names of six parts which were then employed in the dyadic practice at week 1. During the following 3-month practice period, participants met for 13 guided weekly training sessions and could modify their set of six inner parts by replacing old with new ones at any time.

#### Dyadic Practice

Participants performed a daily, 10-min contemplative dyadic exercise throughout the Perspective Module. On the retreat and in the weekly sessions, the perspective dyad was performed face-to-face, while during the practice weeks it was performed online through a voice-based internet-platform that allowed connecting participants with each other (for details, see also Singer et al. 2016). During the dyad, one person took the role of the speaker while the other was the listener; roles were changed after 5 min. First, the speaker’s set of six inner parts was presented to the listener. Then, the speaker chose a recent situation that she experienced and shortly described it from the perspective of one of her inner parts that was randomly selected by a computer algorithm. The listener listened attentively and then guessed which one of the speaker’s inner parts was being voiced. On the side of the speaker, this exercise required imagining a given situation from the perspective of an inner part that was not necessarily active during this situation. Hence, the participant needed to de-identify from the inner perspective that actually was activated during the given situation and take a birds-eye perspective on herself and her inner states. On the side of the listener, the exercise trained mentalizing on others: In order to guess correctly which inner part the speaker was adopting, the listener needed to carefully consider the expressed thoughts and perceptions of the speaker and infer the underlying mental states and beliefs. Taken together, the perspective dyad trained perspective taking on self as well as perspective taking on others, that is ToM.

### Measures

#### Number of Inner Parts

The overall number of different inner parts registered throughout weeks 1–12 of the training was assessed for each participant. Week 13 was not analyzed to avoid interactions with increased demands on the participants due to preparations for the weekend retreat and other assessments. Spelling errors in the inner parts’ names were corrected manually. If two inner part names included the same noun but differed in one adjective, and they did not occur in the same week, they were classified as the same rather than different parts. For example, one participant listed a part called “the tolerant understanding one” and some weeks later a part called “the tolerant one.” The latter was interpreted as an abbreviation of the former and therefore not counted separately.

#### Types of Inner Parts

In addition to the inner parts per se, we were interested in how participants’ inner parts related to the IFS classification (Holmes 2011; Schwartz 1995) and how participants differed concerning these classifications. All identified inner parts were classified according to the IFS.^1^ This expert rating was carried out by the authors of this study, one of which is an expert IFS therapist, Tom Holmes. We employed 12 a-priori defined types described by Holmes (2011) such as *Managers, Protectors or Vulnerable Parts* 2 and added further types post-hoc to increase differentiation. Participants’ inner parts which were defined by positive, happy affect such as “the enthusiastic child” or “the happy princess” were categorized as *Happy Parts* and parts with an optimistic attitude such as “the positive one” were categorized as *Optimists*. The following measures were of interest. First, we assessed each participant’s propensity to identify the different types of inner parts. For each type, the participants’ average numbers of inner parts were determined over the course of weeks 1–12. For instance, participant A’s average set of six parts included three *Optimists* but no *Vulnerable Part,* therefore A’s relative frequency of *Optimists* was .5 (= 3 parts divided by 6) and of *Vulnerable Parts* was 0.

#### Valence of Inner Parts

Finally, we assessed the valence of participants’ inner parts and types of parts. To assess the numbers of positively and negatively valenced parts, three independent raters (one females, aged 22 and 26, one male, aged 30) rated each of the identified parts (*N* = 1147) on a seven-level Likert scale (−3 very negative to 3 very positive). Raters were naïve to the goal of the study. Valence ratings exhibited an excellent internal consistency of Cronbach’s Alpha = .93. A total of 39 parts (only 3.4% of the total amount) were evaluated very ambiguously—with a range of minimum 3–4 points on the valence scale—and were excluded from further valence-oriented analyses. Three measures of valence were derived. First, each participant’s absolute numbers of positive and negative parts were assessed. Secondly, the average valence of parts was determined for each participant. Finally, each of the 14 different type’s mean valence was determined based on the average valence of all inner parts categorized as a respective type. In accordance with the IFS model’s focus on types with extreme affect, we were particularly interested in participants’ propensity to identify very negative and very positive types. We therefore also formed extreme groups including the types with average valences 1 standard deviation above and below all types’ mean (M = .15, SD = 1.40). The negative extreme group covered Worriers, Exhausted Parts, Vulnerable Parts, Fear Parts, and Hurriers. The positive extreme group was comprised of Optimists, Happy Parts, Caring Parts, and Helpers. Each individual’s average frequencies of both extreme groups were determined.

#### Theory of Mind

ToM was assessed using a validated experimental paradigm, the EmpaToM (Kanske et al. 2015), which was specifically designed to measure high-level ToM performance, empathy, and compassion within the same individuals. In this task, participants watched short videos of people talking about autobiographic episodes. ToM performance was assessed by means of multiple-choice questions that required reasoning on the storytellers’ thoughts, intentions, and goals (compared to factual reasoning as control condition). Reaction times (RTs) and error rates were z-transformed, averaged for the ToM condition and combined into one composite measure of ToM performance, which was successfully validated with existing behavioral measures of ToM performance (see Kanske et al. 2015). Change in ToM performance throughout the Perspective Module was assessed by subtracting participants’ performance scores before and after the Perspective Module (see also Trautwein et al. under review). Note that change scores were available for 85% of the total data since calculating participants’ change scores was only possible when both pre- and post-scores were available. For all participants with change scores in ToM performance and inner part data (*N* = 117), the amount of inner parts participants identified throughout the training was correlated with their change in ToM performance (composite of RTs and error rates). In addition, correlations were computed with ToM accuracy scores only (see Trautwein et al. under review).

#### Trait Affect

In order to comprehensively measure participants’ trait affect, we assessed a battery of questionnaires including the Adult Temperament Questionnaire (ATQ; Rothbart et al. 2000; Wiltink et al. 2006), the Beck Depression Inventory (Bech et al. 2001), the personality inventory by Costa and McCrae NEO-PI-R (Ostendorf and Angleitner 2004), the Mental Health Continuum Short Form MHC-SF (Keyes 2009; Lamers et al. 2012), the Positive Affect Negative Affect Scale PANAS (Krohne et al. 1996; Watson et al. 1988), the Short Affect Intensity Scale SAIS (Geuens and Pelsmacker 2002), and the Types of Positive Affect Scale TTPAS (Gilbert et al. 2008). Principal component analysis was used to reduce the complexity of the data revealing three factors: Negative Affect, Positive Affect, and Low Arousal Positive Affect (see Singer et al. 2016, Supplement S1).

#### Motivation

In order to be able to control for participants’ motivation to do the dyadic practice, this measure was assessed after the completion of the Perspective Module, using a custom-made questionnaire. Specifically, participants were asked to retrospectively rate their liking of the dyadic practice, how much they looked forward to doing the practice, and how well they could integrate the practice into their daily lives on a five-level Likert scale.

## Results

### General Descriptives of Inner Parts and Classification into Types

#### Number of Parts

**Fig. 2:**
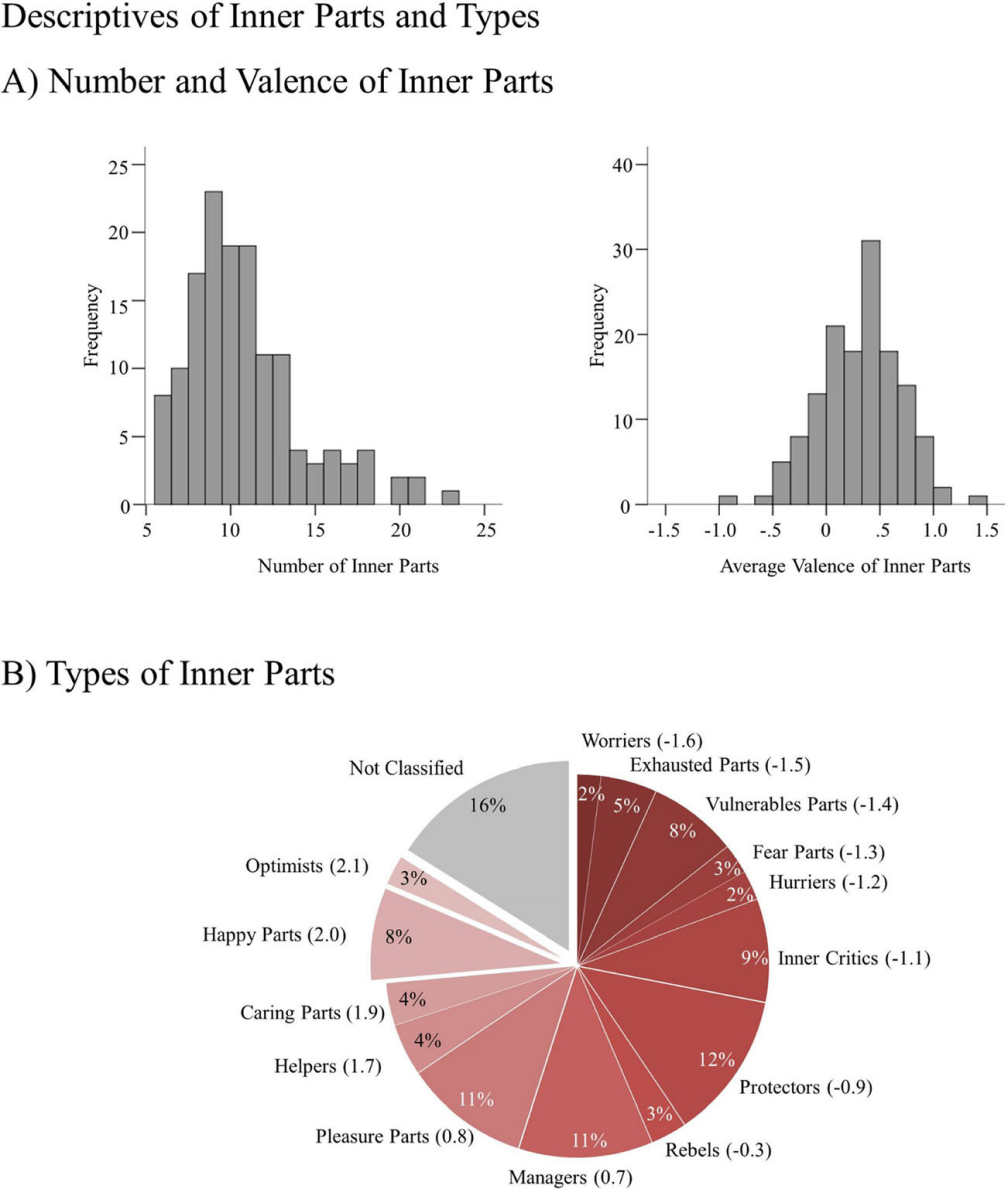
**a** Overall numbers of inner parts identified by participants during the 12 weeks of the training are displayed in the *left graph*. The *right graph* shows participants’ average valence of all identified parts. **b** Relative frequencies of identified types of inner parts. Types are arranged and colored according to their average valence (*in parentheses*)

The distribution of number and valence of the identified parts is displayed in Fig. 2, panel a. In total, participants identified 1147 inner parts. Each participant identified on average 11 inner parts (range, 6 (required minimum) to 23; SD = 3.45) over the course of the training.

#### Types of Parts

Seventy-four percent of the identified parts could be clearly classified into the typical categories of types suggested by Holmes (2011). As depicted in Fig. 2, panel b, *Protectors* were the most frequent types (12%), followed by *Managers* and *Pleasure Parts* (both 11%), *Inner Critics* (9%), and *Vulnerable Parts* (8%). Among the less frequent types were *Exhausted Parts* (5%), *Caring Parts* and *Helpers* (both 4%), next to *Fear Parts* and *Rebels* (both 3%). The least frequent types were *Hurriers* and *Worriers* (2%). The inner parts that could not be classified as prototypical types were classified using our additional categories. Among the additional types, *Happy Parts* were the most prominent (8%), followed by *Optimists* (3%).

#### Valence of Parts

Participants identified on average 4.89 negative and 6.21 positive inner parts (negative parts’ range, 1 to 11; SD = 2.15; positive parts’ range, 2 to 17, SD = 2.51). The average valence of parts participants identified exhibited a slight positivity bias with a population mean of .31 (range, −.86 to 1.43; SD = 0.38) on a seven-level Likert scale ranging from −3 to +3. In terms of extremely valenced types, participants identified on average 1.16 inner parts belonging to the five highly negative types (range, 0 to 3, SD = .70) and 1.10 inner parts belonging to four highly positive types (range, 0 to 2.83, SD = .66).

### Correlation Between Valence of Inner Parts and Trait Affect

In order to test whether the valence of the identified inner parts could be validated by more established measures of trait affect, participants’ scores on trait affect factors were correlated with (a) the average valence of participants’ identified inner parts and (b) the number of parts belonging to the “extremely valenced types” (most positive types accounting for 19% of the totality of parts, most negative types accounting for 21% of the totality of parts).

#### Average Valence

The valence of participants’ parts was significantly and positively correlated with scores on the factor Positive Affect (*r* = .27, *p* = .001), thus, the more positively valenced the inner parts of a participant were, the more positive trait affect was reported. Correlations with factor scores on Negative Affect (*r* = −.14, *p* = .096) and Positive Affect Low Arousal (*r* = .15, *p* = .076) were not significant.

#### Number of Extremely Valenced Types

**Fig. 3:**
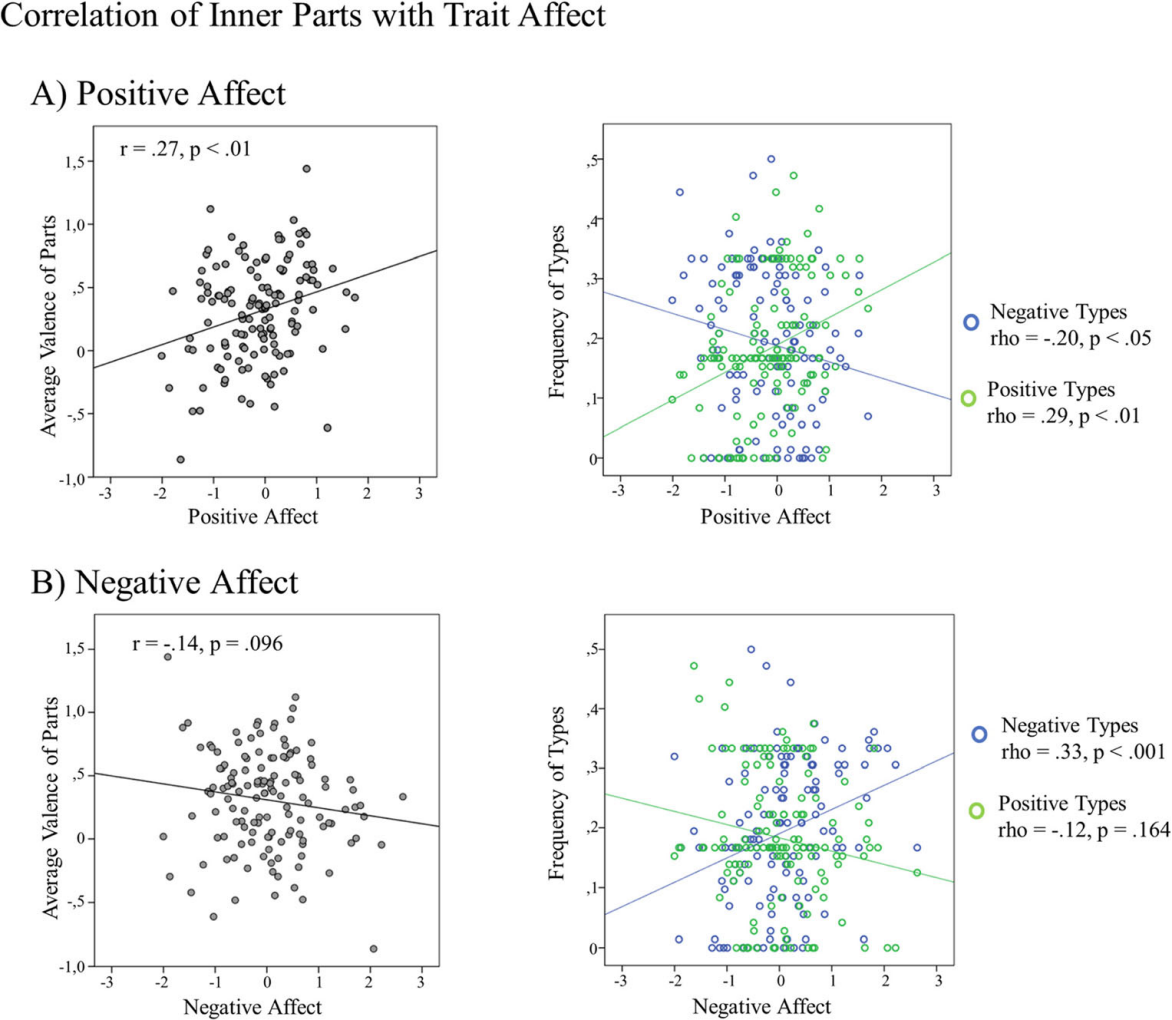
Correlations of the valence of inner parts (*left graphs*) and the relative frequencies of extremely valenced types of inner parts (*rights graphs*) with trait affect. **a** The relations for positive affect. **b** The relation for negative affect. Negative types are depicted in *blue*; positive types are depicted in *green*

Due to non-normality of the distribution of numbers of extremely valenced types, Spearman correlation coefficients are reported in the following. Participants with more inner parts belonging to the extremely positive type category also exhibited higher scores on the factors Positive Affect (rho = .29, *p* = .001) and Positive Affect Low Arousal (rho = .23, *p* = .007) (see Fig. 3). In addition, participants with a higher number of the most negative types scored higher on the factor Negative Affect (rho = .33, *p* < .001) and lower on the factor Positive Affect (rho = −.20, *p* = .023). No other correlations were significant (*p*s > .16) (see Fig. 3).

Taken together, findings show a coherent relation between the valence attributed to the inner parts and the dispositional affect of participants assessed with psychological trait questionnaires. Hence, the affective pattern revealed by participants’ inner parts was mirrored by established assessments of positive and negative trait affect.

### Correlation Between Number of Inner Parts and Change in Theory of Mind Performance

**Fig. 4:**
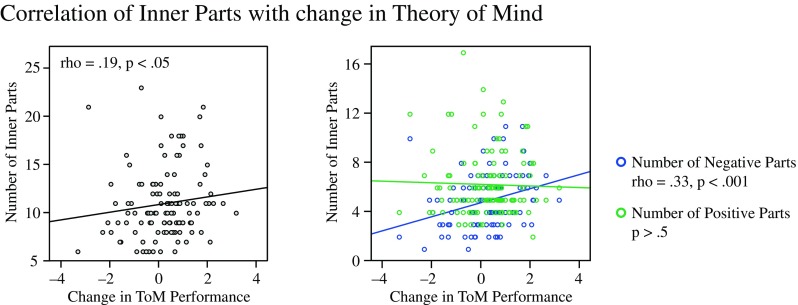
Correlations of the number of inner parts identified by participants and their improvement in ToM performance. Correlations with negative parts are depicted in *blue*, correlations with positive parts are depicted in *green*

The main goal of the present study was investigating whether individual differences in improvements in ToM performance elicited by the Perspective Module (Trautwein et al. under review) were related to differences in the number of inner parts participants identified during the course of the training, that is, the depth of participants’ inner part work. Spearman correlation coefficients are reported due to the non-normal distribution of the variable number of participants’ inner parts. Indeed, participants’ improvement in ToM performance during the Perspective Module was significantly correlated with the overall number of identified parts (rho = .19, *p* = .046). Hence, the more inner parts participants identified during the 12 weeks of training, the more they improved in understanding others’ mental states. Interestingly, the valence of the identified parts was crucial. While identifying and differentiating between a greater number of *negative* inner parts correlated significantly with improvements in ToM performance (rho = .33, *p* < .001), no relation to the amount of *positive* parts was found (rho = .04, *p* = .676) (see Fig. 4). When controlling for potential confounding variables such as differences in baseline ToM performance (ToM performance before the Perspective Module) and differences in baseline Negative Affect, the number of negative parts remained a stable predictor of change in ToM (*r* = .34, *p* = .000; *r* = .26, *p* = .005). Also, when controlling for participants’ motivation to do the perspective dyad (mean = 3.68 (ranging from a minimum of 1 to a maximum of 5), SD = 1.14), the correlation between number of negative parts and change in ToM remained significant (*r* = .28, *p* = .003). Finally, we analyzed the relation between inner parts and ToM accuracy (see Trautwein et al. under review). This analysis similarly revealed that improvements in ToM accuracy were related to the amount of identified negative inner parts (rho = .20, *p* = .030), while no relation was found for positive parts (rho = −.03, *p* = .714) or total number of parts (rho = .09, *p* = .333).

In sum, these findings suggest that individual differences in the propensity to identify inner parts, particularly negatively valenced inner parts, over the course of the training were predictive of individual differences in the ability to accurately understand intentions and beliefs of other people after 3 months of training.

## Discussion

Putting ourselves in the shoes of others is a crucial socio-cognitive skill that is required not only in our everyday social life, but also with regard to cross-cultural understanding and mastering global political challenges (Batson et al. 1997; Galinsky and Moskowitz 2000; Paal and Bereczkei 2006). Only recently, studies have shown that adults’ capacity to represent and infer others’ mental states can be enhanced through contemplative mental training programs (Mascaro et al. 2013; Schurz et al. 2014; Trautwein et al. under review). The processes that facilitate these improvements are yet unknown, however. We investigated whether understanding other people may be inherently linked to understanding oneself. Having more complex and fine-grained knowledge about one’s own emotional, cognitive, and behavioral facets is a useful capacity in everyday life and therapeutical settings (Holmes 2011; Schwartz 1995) and may help to better understand the mental states of others. Accordingly, the main goal of the present study was to investigate whether learning to better understand oneself is related to improvements in the cognitive understanding of others.

This question was addressed in the scope of a longitudinal mental training study (Singer et al. 2016), the *ReSource Project*, in which a large representative participant sample underwent a 3-month Perspective Module, in which metacognitive abilities and perspective taking on self and others were practiced. Specifically, participants performed a daily 10-min dyadic exercise with another person and practiced both identifying and taking the perspective of inner personality parts rooted in inner parts work of the IFS model (Holmes 2011; Schwartz 1995) and taking the perspective of another person (Kok and Singer 2017).

Results revealed that (a) participants identified inner parts that could largely be classified according to the established typology used in IFS (Holmes 2011; based on Schwartz 1995), (b) the affective valence of participants’ identified inner parts could be validated with established measures of dispositional trait affect, and (c) learning to identify and discern between greater numbers of inner parts, especially negatively valenced parts, was associated with greater improvements in ToM capacity after the mental training. Note that the assessment of ToM performance with a laboratory task (Kanske et al. 2015) was completely independent in content and time of assessment from the daily mental exercises practiced during the Perspective Module.

Since IFS is typically used in therapeutic contexts (Holmes 2011; Schwartz 1995; Shadick et al. 2013) rather than in psychological empirical research, we first aimed at investigating several fundamental features of participants’ inner parts, such as how many and which types were identified in the course of the study. On average, participants reported 11 distinct inner parts throughout the perspective training. The majority of inner parts identified by our participant sample could be categorized according to the typology described by Holmes (2011). Hence, the Internal Family Systems model which originated in psychotherapy (Schwartz 1995) could account well for cognitive and affective patterns exhibited by healthy adults ranging from 20 to 55 years. Furthermore, we could validate our new approach with prototypical psychological trait questionnaires. Individuals who identified a larger amount of strongly negative types of parts during the Perspective Module also reported more negative trait affect and, accordingly, individuals who identified a higher number of positive inner parts had a higher disposition for positive affective states as measured with classical trait questionnaires. Similarly, the more positive the average valence of participants’ inner parts was, the more positive trait affect they reported. This coherent correlation pattern between types of identified inner parts during daily contemplative exercise and widely used psychological dispositional affect measures supports the view of inner parts as valid constructs in the assessment of individual differences in personality. Future research may focus on the motivational foundations of inner parts and the relationship between inner parts to other motives, personality traits, and attitudes.

Most importantly, the finding of a significant correlation between the number of participants’ inner parts identified throughout the 3-month training and their improvements in high-level ToM performance clearly suggests that the degree of familiarization with one’s own internal dynamics and affective and cognitive patterns is linked to improvements in understanding the mental states of other people. This interplay between taking one’s own and others’ perspectives is in line with neuroimaging studies reporting a neural overlap between brain regions that are reliably activated during both processes (Lombardo et al. 2010; Mitchell and Phillips 2015) and mimics similar theories in the domain of affective empathic responses (Singer 2012; Lamm and Singer 2010) that show a relationship between the degree of understanding ones’ own emotions and the degree of empathizing with others’ affective states (Bird et al. 2010; Lamm and Singer 2010; Steinbeis and Singer 2014). Our findings further add to an idea put forward by Dimaggio et al. (2008) that better self-reflection can lead to greater awareness of others. Another important theoretical framework based in philosophy (Goldman 1995a, b) and later developed by neuroscientists (Gallese and Goldman 1998) has been the simulationist account which posits that our capacity to understand another’s mind relies on our privileged access to our own mental states (Goldman 2006). In accordance, becoming aware of a greater variety of our own different inner parts associated to patterns of affective, bodily, and cognitive states may increase our ability to identify similar mental states in other people.

Finally, we were interested in how the valence of identified inner parts was related to improved ToM performance. While improvements in mentalizing on others were generally related to identifying a high number of parts, this correlation was driven by the amount of negatively valenced parts and absent for positive inner parts. This result is in line with IFS theory, suggesting that vulnerable, anxious, or doubtful affective states are more difficult to become aware of in one’s own experience, because seeing them requires overcoming an initial resistance against these painful affects (Holmes 2011; Schwartz 1995). Accordingly, participants who identified a greater amount of negative parts throughout the training may have engaged in a more extensive search of their inner parts, even the difficult ones, and thus engaged in the inner part work more thoroughly, dedicating more energy and attention to their practice. Clinical psychology has generally regarded accepting—as opposed to avoiding—negative emotions as beneficial for mental health (Davis et al. 2015; Williams and Lynn 2011). According to the IFS model, accepting previously unacceptable parts of oneself and others plays a central role for the therapeutic success (Schwartz 2013). Interestingly, experiential avoidance—conceptualized as the opposite of acceptance (Williams and Lynn 2011)—has been found to involve reduced ToM capacities in psychiatric patients (Vanwoerden et al. 2015). Vanwoerden and colleagues argued that an unwillingness to contact uncomfortable experiences may result in a lack of integrated memories of negative states which may, in turn, disable the attribution of such negative states in others. Similarly, participants who accepted and allowed uncomfortable experiences in themselves may have increased their differentiation between negative mental states, which allowed for a better understanding of others’ mental states.

In the following, we will discuss in more detail the potential mechanisms that may underlie the relation between inner parts work as an example of understanding oneself and the enhanced socio-cognitive capacity to understand others. Firstly, one contributing factor may be participants’ discovery of affective and cognitive mental patterns that formerly had not been accessible or represented. This recognition and forming of more elaborated representations of diverse mental patterns may have enabled the more differentiated attribution of similar patterns to others. During the training sessions, self-exploration was explicitly encouraged by targeted exercises and participants were asked to detect new inner parts in their experience of themselves both during the exercises and at home (Singer et al. 2016). As the positive correlation between the number of parts identified during training and increased ToM performance after the training suggests, the discovery of new self-aspects, and particularly aspects that may seem difficult at first, plays a key role in the ability to understand other people’s mental states.

Secondly, participants may have arrived at generally enhanced attention to and processing of mental states per se. To date, all training programs which could successfully improve ToM performance have in some form guided participants to focus their attention on mental states (e.g., by discussing these phenomena in a group or with a trainer; see Cavallini et al. 2015; Lecce et al. 2014a, b). Similar processes may be elicited by inner parts work, which explicitly emphasizes to focus on mental states as well as their fluidity.

Thirdly, as Schwartz (1995) spells out, learning about the concept of inner parts fosters a view of self and other not as single units but as multiplicities of aspects. This view may reduce the habitual tendency to perceive others as overly consistent across varying situations (Sande et al. 1988) and thereby aid in increasing flexibility and accuracy in the understanding of the complexity of other persons’ different inner mental states.

Fourth, the improvement of ToM performance may be specifically boosted by the contemplative perspective dyad in which participants trained to willfully identify with an inner part and then take the perspective of that inner part to recount a certain situation from the past day (Kok and Singer 2017). In so doing, participants needed to decouple from the situation how it was actually experienced as well as from the real inner part(s) that was active in this situation and recount it from the perspective of another part, possibly contrary in style from the one that had been actually prevalent in the given situation. As an example, the aversive situation of receiving a parking ticket had to be told from the happy inner child’s perspective. Such “decoupling” from experienced reality has been advocated to be a basic process needed for taking the perspective of another person (Frith and Frith 2003). Accordingly, participants who came up with more inner parts also trained to take the perspective on a greater variety of different inner parts in unusual contexts, and got better in decoupling. Future studies could help identify whether neuronal networks underlying individual differences in ToM performance are shared with those underlying individual differences in identifying and taking the perspective of self-related inner parts as practices in the present study. In addition, to conclusively describe the (combination of) processes that underlie the beneficial effect of learning to understand oneself for understanding others, future studies will need to perform more selective manipulations, for instance by separately training the decoupling from a given situation or viewpoint on the one hand and the more elaborate understanding of internal dynamics among inner parts in self and others on the other hand.

In sum, the present study clearly suggests that inner parts work and training to flexibly take perspective on self-related inner mental states is not only promising in therapeutic settings, but also in non-clinical settings aiming to foster psychological health and social intelligence as well as for fundamental research in the fields of personality and social psychology and social neurosciences. We demonstrated that healthy adults between 20 and 55 years could easily identify prototypical inner parts such as “the inner manager” or “the inner child.” The assessment of these inner parts was further validated on the basis of participants’ affective personality styles as measured through a battery of classical psychological trait measures. Importantly, learning to identify a larger variety of internal personality aspects was predictive of training-related improvements in one of the most critical interpersonal skills—flexibly and accurately inferring and representing other people’s mental states, that is ToM performance. Interestingly, especially learning to identify negative aspects of the self was predictive of a better understanding of other people. This insight could prove important in an increasingly complex and interconnected world where taking the view of others, especially those from different cultures or with different religious backgrounds, becomes ever more difficult—and ever more necessary. Targeted trainings that aim at improving people’s capacity to understand themselves and put themselves more easily in the shoes of others could help fostering the care and support that is needed to deal with the global challenges of today.

## Electronic supplementary material

ESM 1 (DOCX 17 kb)

ESM 2 (PDF 873 kb)

## Footnotes

1 In cases of ambiguous parts, nouns were given priority to adjectives. For example, for the classification of “the ambitious counselor,” “counselor” was emphasized.

2 Note that for our non-clinical population, we employed Holmes’ type of *Vulnerable Parts* instead of Schwartz’ type of *Exiles.*
