# Supplementary material for: Know Thy Selves: Learning to Understand Oneself Increases the Ability to Understand Others
Source: J Cogn Enhanc. 2017 May 16;1(2):197–209. doi: 10.1007/s41465-017-0023-6 (PMC7089715; doi:10.1007/s41465-017-0023-6)
Supplement: Supplementary file 1 — (DOCX 17 kb) [file 41465_2017_23_MOESM1_ESM.docx]

**Supplementary Material to**

**Know thy Selves:**

**Learning to understand oneself increases the ability to understand others**

Supplement Table S1: Exploratory Factor Analysis (PCA). Factor loadings of the measures of affective dispositions (Pattern matrix).

| **Measures** | **F1** | **F2** | **F3** |
| --- | --- | --- | --- |
| *Factor 1: Positive Affect* (35% variance) |  |  |  |
| NEO_PIR_positive_emotion | **.866** | .032 | -.082 |
| ATQ_positive_affect | **.761** | -.064 | .047 |
| NEO_positive_affect | **.759** | -.050 | .122 |
| MHC_EWB | **-.719** | .045 | -.109 |
| NEO_PIR_warmth | **.675** | .143 | -.151 |
| PANAS_positive | **.635** | -.240 | -.115 |
| TTPAS_warmth | **.613** | .031 | .557 |
| TTPAS_active | **.556** | -.193 | -.539 |
| *Factor 2: Negative Affect* (16% variance) |  |  |  |
| ATQ_negative_affect | .047 | **.947** | .017 |
| ATQ_fear | .056 | **.911** | .199 |
| ATQ_sadness | .098 | **.740** | -.046 |
| NEO_negative_affect | -.209 | **.688** | -.058 |
| ATQ_frustration | .174 | **.542** | -.146 |
| BDI_affective | -.378 | **.394** | -.064 |
| PANAS_negative | -.333 | **.307** | -.208 |
| *Factor 3: Serenity* (8% variance) |  |  |  |
| TTPAS_relaxed | .091 | -.076 | **.773** |
| SAIS_serenity | -.407 | -.216 | **.509** |
