# Supplementary material for: Know Thy Selves: Learning to Understand Oneself Increases the Ability to Understand Others
Source: J Cogn Enhanc. 2017 May 16;1(2):197–209. doi: 10.1007/s41465-017-0023-6 (PMC7089715; doi:10.1007/s41465-017-0023-6)
Supplement: Supplementary file 2 — (PDF 873 kb) [file 41465_2017_23_MOESM2_ESM.pdf]

Specific Benefits of Mental Training Types for Attention, Compassion, and Theory of Mind

Authors: Fynn-Mathis Trautwein[1], Philipp Kanske[1], Anne Böckler[1,2], Tania Singer[1]*

Affiliations:

[1]Department of Social Neuroscience, Max Planck Institute for Human Cognitive and Brain Sciences, 04103 Leipzig, Germany.

[2]Department of Psychology, Würzburg University, 97070 Würzburg, Germany.

*Correspondence to: singer@cbs.mpg.de.

In a more and more complex and interconnected world—with constant exposure to multi-channel online stimulation, global competition for limited resources, and penetration of socio-cultural borders—the question if and how human capacities such as attention and social and emotional intelligence can be cultivated has become increasingly salient. Here, meditation-based mental training might represent an effective means to induce plasticity in relevant cognitive, affective, and social functions[1-3]. However, previous research focused on interventions that integrate a range of different contemplative practices, such as on the well-known mindfulness-based stress reduction program (MBSR)[4], and mostly lacked the direct comparison with other meditation-based control conditions. Therefore, it remains unclear whether different types of practice, pursuing different aims[5], can induce plasticity in distinct mental functions. In a large-scale longitudinal study, the ReSource Project (N = 332), we show that three consecutive three-month mental training modules, each consisting of different practices aimed at cultivating either present-moment attention, socio-affective qualities such as compassion, or socio-cognitive skills such as perspective taking on self or others, have largely differential and specific effects on increasing the respective targeted abilities. While attention performance improved more generally across different training modules, compassion increased most strongly after socio-affective training and theory of mind showed selective improvements after socio-cognitive training only. These results reveal evidence for plasticity in socio-emotional and -cognitive intelligence in healthy adults and further illustrate that specific mental training practices are needed to induce benefits in different domains of mental functioning, providing a foundation for evidence-based development of more targeted interventions adapted to the needs of different education, labor, and health settings.

Soaring research into secularized meditation-based mental training interventions, which aim at promoting wholesome qualities of mind and heart, has yielded promising findings[1,2]. However, stringent evidence for effectiveness and specificity of training induced plasticity of mental functions such as attention, social emotions, and social cognition requires a study design that implements different types of contemplative practices[5] within structurally equivalent intervention conditions (e.g. with respect to setting, teachers, and amount of training). To this end, we designed a large-scale longitudinal mental training study, the ReSource Project[6], implementing three consecutive three-month mental training modules (Presence, Affect, Perspective) consisting of distinct types of contemplative practices and targeting attentional-, socio-cognitive, or socio-affective skills (Figure 1). The Presence Module focused on cultivating present-moment attention and interoceptive awareness through exercises (e.g. Breathing Meditation and Body Scan) typically employed in other mindfulness-based interventions such as MBSR[4]. The Affect Module focused on cultivating affective qualities of care, gratitude, and loving-kindness, as well as dealing with difficult emotions through acceptance and increasing prosocial motivation. The Perspective Module focused on improving metacognitive awareness of thoughts and perspective taking on self and others (the latter also referred to as mentalizing or theory of mind; ToM)[7,8].

Here, we focused on three target measures, namely behavioral markers of attention (Figure 2a), compassion, and ToM (Figure 2b), as we expected the three modules to have differential effects on these specific outcomes. Thus, we expected the Presence Module to improve attention based on both a) its focus on increasing present-moment attention and b) previously reported effects of similar mindfulness interventions on attention[9-11]. An open question is, however, whether attention is also enhanced by other types of mental practices, for example those focused on

loving-kindness[12], that do also (implicitly) demand self-regulation of attention. Second, based on its focus on cultivating social emotions, we expected the Affect Module to particularly increase compassion. Accordingly, previous intervention studies on compassion and loving-kindness meditation provided evidence for increased positive affect and concomitant activation increases in neuronal networks associated with positive emotions, affiliation, and care[13-16]. Moreover, it has been debated how exactly ethical-motivational qualities such as compassion are linked with mindfulness[17-19]; and thus it is unclear whether such qualities are cascade-like outcomes of present-moment awareness and attention focused practices as implemented in the Presence Module—and would therefore also be enhanced by this Module alone . Third, we expected the Perspective Module to be particularly efficient in enhancing ToM. The module includes practices explicitly targeting this intersubjective skill and is based on evidence of distinct neural networks supporting ToM vs. empathy and compassion[20]. While some studies have associated mindfulness with a shift in the perspective on one's own experience[21], to date little is known about whether (and which) contemplative practices foster perspective taking on others. More general meditation effects on social cognition have been assessed using the Reading the Mind in the Eyes Test—a task that has recently been shown to measure emotion recognition, rather than ToM[22]—however with heterogeneous results[23,24]. Thus, it remains unknown whether and which type of mental training can actually improve ToM accuracy. Such evidence would be highly informative for the design of interventions for a range of clinical conditions with ToM impairments[8].

The study design (Figure 1) allowed us to assess differential effects of the three training modules in a well-controlled design by comparing pre-post effects of each module against a retest cohort and against the other training modules, acting as active controls. Participants were randomly assigned to one of three training cohorts (TC1, TC2, TC3) or a retest control cohort (RCC). TC1

and TC2 first completed the Presence Module, followed by Affect and Perspective in reverse orders. TC3 only completed the Affect Module to serve as an active control condition for Presence. The modules consisted of an introductory three-day intensive retreat, weekly two-hour group sessions, and daily practice completed via web platforms. Exercises are illustrated in Figure 1b (see Supplementary Methods for a detailed description of the training modules).

First, we tested whether the different training modules have effects on the respective targeted outcome measures (Presence on attention, Affect on compassion, and Perspective on ToM) over and above effects of retest and the other training modules. Mean scores of each measure at specific time points (Figures 2c, 2d, 2e) were used to compute difference scores for each time interval. Separate linear mixed models for each outcome measure contained fixed effects for *module* (retest, Presence, Affect, Perspective) and *interval* (3 levels: T0 to T1, T1 to T2, T2 to T3). In case of a significant *interval* by *module* interaction, we also tested effects for individual time intervals. Finally, we also explored module effects not specified by our hypotheses using additional contrasts.

For training module effects on attention (Figure 2f), we analyzed an index of general attentional capacity from the cued flanker task, which subsumes executive control and reorienting of attention (i.e. accuracy in invalidly cued, flanker-target conflict trials, see methods and Trautwein et al., 2016[25], for details). As predicted, the Presence Module differed from retest ($b = 0.437$, $z = 3.856$, $p < .001$). However, no specific effect was found when comparing Presence and Affect at T0 to T1 ($b = 0.035$, $z = 0.292$, $p = .77$). A significant interaction between *interval* and *module*, $X^2(3) = 12.055$, $p = .007$, indicated that module effects depended on the time interval and additional comparisons for individual intervals revealed that Affect also differed from retest at

T0 to T1 ($b = 0.403$, $z = 3.013$, $p = .003$), but none of the comparisons after T1 were significant (all $p > .14$). Thus, the interaction reflects that Affect was effective when applied as a first training module, whereas no further improvements were induced when applied after the Presence Module. The effect sizes for the observed reliable improvements after the three-month modules of Presence and Affect from T0 to T1 were small in size (see Supplementary Table S7).

These improvements were found for an index of a general attentional capacity, which subsumes executive control and reorienting of attention[25]. Supplementary analyses of isolated markers of these components of attention showed that improvements were driven by the executive control component; furthermore, improvements after Affect and Presence were not related to changes in response time (used as covariate), however, improvements after Affect were accompanied by an increase of mean response times (see Supplementary Results).

In sum, a general improvement in attentional performance occurred after the first three-month training module, irrespective of whether it was Presence or Affect, while additional training through Affect or Perspective Modules did not lead to further improvements.

To assess training effects on compassion, we analyzed trial-wise ratings of compassion in the EmpaToM task[26,27], which presents short videos of people describing neutral or emotionally negative live events (see methods for details). The daily practice of loving-kindness meditation aims at developing unconditional love, care, and kindness towards others, a quality that is paralleled by the feeling of compassion when confronted with suffering[12]. Because the definition of compassion given to participants (see methods) focused on the general positively valenced feeling of warmth, kindness, and concern and did not explicitly constrain it to situations of suffering, we expected a general increase in compassion across neutral and emotional videos, in line with previous empirical findings[14,15].

Overall contrasts of module effect estimates (Figure 2g) across respective time intervals showed larger effects for Affect as compared to retest (*b* = 1.027, *z* = 4.420, *p* < .001), Perspective (*b* = 0.389, *z* = 1.973, *p* = .049) and Presence (*b* = 0.292, *z* = 2.542, *p* = .011). Furthermore, there was a significant interaction of *interval* and *module*, $X^2(3) = 9.604$, *p* = .022, indicating that effects depended on the order of the training modules. Descriptively (Figure 2d), compassion showed stronger increases after each Affect compared to the respective Perspective and retest intervals, however, the effect of Affect was most pronounced from T1 to T2 for TC1, the cohort that completed Affect before Perspective. Comparing effects between groups at individual time intervals revealed that, at T1 to T2, differences between Affect (TC1) and retest were significant (*b* = 0.677, *z* = 4.992, *p* < .001), but did not reach significance at T0 to T1 for TC3 (*b* = 0.129, *z* = 0.985, *p* = .325) or T2 to T3 for TC2 (*b* = 0.22, *z* = 1.626, *p* = 0.104). Similarly, at T1 to T2, differences between Affect (TC1) and Perspective (TC2) were significant (*b* = 0.319, *z* = 2.286, *p* = .0223), but not at T2 to T3 when both cohorts had switched modules (*b* = 0.070, *z* = 0.503, *p* = .615). Finally, Perspective also differed from retest overall (*b* = 0.508, *z* = 2.631, *p* = .008) and at T1 to T2 in TC2 (*b* = 0.359, *z* = 2.610, *p* = .009), but not at T2 to T3 in TC1 (*b* = 0.150, *z* = 1.103, *p* = 0.27).

Effect sizes for the Affect Module increase in compassion were negligible at T0 to T1 (i.e., for the first three-month training in TC3), but large at T1 to T2 (for TC1 doing the Affect Module after the Presence Module), and small at T2 to T3 (for TC2 doing Affect after Presence and Perspective), while Perspective had a medium effect at T1 to T2 (in TC2) and a negligible effect at T2 to T3 (in TC1) (see Supplementary Table S7).

Additional follow-up analyses were done separately for ratings from neutral and emotional conditions. These analyses largely replicated the main analysis, with significant effects of Affect

in all comparisons apart from the comparison with Perspective in the emotional condition (see Supplementary Results).

In sum, results are consistent with the hypothesis that Affect has the strongest effect on compassion, beyond retest, Presence and Perspective. These effects were mostly driven by TC1, potentially indicating that the module's effectiveness depended on the order of training modules. Because the Perspective Module also led to increases in compassion, these improvements in TC2 after Perspective may have limited the effect that subsequent Affect training could still have on our measure of compassion.

To test for training related improvements in cognitive perspective taking, we evaluated accuracy in ToM questions of the EmpaToM task. Overall contrasts of module effects (Figure 2h) showed that Perspective was accompanied by a stronger increase in ToM performance compared to the respective retest intervals ($b = 0.53$, $z = 2.032$, $p = .042$) and compared to Affect ($b = 0.58$, $z = 2.196$, p = .028). No significant differences were found for additional comparisons not involving the Perspective module (i.e. between Presence, Affect, and retest) (all $p > 0.57$). For ToM performance, module effects did not depend on measurement interval, $X^2(3) = 0.219$, $p = .974$). The effect sizes for the reliable improvements in ToM performance after the respective three-month Perspective Modules were small in size (see Supplementary Table S7).

Supplementary analyses indicated that these improvements in ToM accuracy were independent from changes in RT and largely replicated for a composite score of accuracy and RT (as also reported in previous studies[26,27]). Furthermore, no training related changes were observed in control questions, suggesting that the improvements in ToM performance were really specific to understanding beliefs and intentions of other people rather than reflecting an increase in general cognitive capacity (see Supplementary Results).

In sum, training-related improvements of ToM performance were specifically induced by the Perspective Module irrespective of when this module was implemented in the training sequence. Note that, descriptively, all groups improved between T0 and T1 (see Figure 2e), however there were no significant differences between retest and any of the training groups suggesting that these changes are retest effects, that is to improvements due to performing the task the second time.

As another test of the specificity of the three different training modules, we compared the respective effects of a given module across all three outcome measures (Figure 3), hypothesizing that the largest effects would be found within the outcome measure that was the a-priori target of a given module, that is, we expected strongest effects of Presence for attention, of Affect for compassion, and of Perspective for ToM measures. Importantly, we focused on changes contrasted against retest, because change might differ between measures not only due to specific module effects but also due to susceptibility to retest effects. Separate mixed models were estimated for each training module (Presence, Affect, Perspective) with fixed effects for *intervention* (one of the modules vs. retest) and *outcome* (attention, compassion, ToM).

For Presence, a significant interaction of *intervention* and *outcome*, $X^2(2) = 9.569$, $p = .008$, indicated that the module's effects depended on the outcome. Linear contrasts revealed that training-related changes for Presence vs. retest were larger for attention as compared to compassion ($b = 0.599$, $z = 3.051$, $p = .002$) and ToM ($b = 0.401$, $z = 2.042$, $p = .041$). Presence vs. retest differences did not differ for compassion and ToM ($b = -0.198$, $z = -1.044$, $p = .296$). Thus, the Presence Module seems to be most efficient in increasing attention as compared to its effects on the other two dependent measures, compassion or ToM.

For Affect, a significant interaction of *intervention* and *outcome*, $X^2(2) = 9.809$, $p = .007$,

indicated dependency of the module's effects on the outcome. Linear contrasts revealed that differences in training-related changes of the Affect Module vs. retest were larger for compassion as compared to attention ($b = 0.263$, $z = 2.056$, $p = .04$) and ToM ($b = 0.375$, $z = 3.056$, $p = .002$), while there was no difference between attention and ToM ($b = 0.111$, $z = 0.866$, .386). Again, these findings are in line with our hypothesis that the Affect Module should be most efficient in boosting compassion as compared to attention or ToM.

Descriptively, the Perspective Module had the strongest effects on ToM performance and on compassion. However, there was no significant interaction between *intervention* and *outcome*, $X^2(2) = 3.028$, $p = .386$, and none of the specific comparisons were significant (all $p > .119$). Thus, while Perspective was the only module that led to a significant increase in ToM performance, this effect was statistically not significantly larger than the module's effect on the other outcome measures.

The present results show that daily contemplative mental training performed over several months can indeed induce plasticity in attentional, socio-affective, and socio-cognitive functions: All training modules boosted the respective target outcome beyond changes in the retest control group, with effect sizes ranging from small to large depending on the sequence of a given practice type in the entire nine-month longitudinal ReSource study[6]. Moreover, the results decompose the broadly used concepts of meditation and mindfulness by directly demonstrating that different types of contemplative practices, often subsumed within a single program[4,23,28], have specific effects on the mental faculties of attention, compassion, and ToM. Whereas attention was boosted by both, the Presence and Affect modules, compassion was most effectively increased by the Affect, and ToM performance only by the Perspective Module.

For the Presence Module focusing on cultivation of present-moment attention and interocepetive awareness through practices such as Breathing Meditation and Body Scan—which is thus most similar to the well-known MBSR program[4]—results are in line with previous findings of improved attention after mindfulness-based interventions[9-11]. Interestingly, these effects were restricted to attention, as the Presence Module neither increased compassion nor ToM, that is, social capacities were not affected by these types of basic attention focused mindfulness practices, speaking against cascade-like models of mindfulness and emphasizing the need to explicitly cultivate intersubjective, compassion-based and ethical qualities[17-19]. Attention, however, was similarly augmented by the Affect Module—which was also the most efficient module in increasing compassion. This finding suggests that in addition to targeting socio-emotional and motivational processes, practices of the Affect Module also foster attention. And indeed, the requirement of Loving-kindness Meditation[12], a core practice in the Affect Module as well as in other compassion-based intervention programs[19,28], is to maintain a stable focus on a mental image (e.g., of a close person) while generating motivational states of loving-kindness. Furthermore, Affect did not have an additional effect on attention when practiced after the three-month Presence Module, thus future research may explore whether such effects of longer training durations would occur in more difficult measures of attention performance.

The finding of different ways to foster attention bears relevance for the treatment of a range of psychiatric disorders with deficiencies in attention[3]. For example, the practices of the Presence Module might be suitable for children and adults suffering from ADHD, while practices fostering positive affect and at the same time enhancing attention—as those in the Affect Module—might support treatment of affective disorders that are characterized by both, emotion and attention regulation difficulties[29].

The finding that the Affect Module led to strongest improvements in compassion—assessed as experienced feelings of care, warmth and benevolence—extends previous studies on kindness-based meditation showing increased positive affect[13-16]. Importantly and in contrast to previous studies, participants were not instructed explicitly to apply the learned skills when performing the EmpaToM task, suggesting that the present findings represent trait changes in the tendency to spontaneously experience compassion for others. The finding that present-moment and attention-based mindfulness practices as taught in the Presence Module alone did not increase compassion suggests that explicit cultivation of intersubjective qualities such as empathy, gratitude, loving-kindness, and prosocial motivation is advisable to foster compassion. This is in line with the hypothesis that compassion is rooted in a care and affiliative, other-related motivational system[14,15,30,31]. Interestingly, although the Affect Module was most efficient in boosting compassion, the Perspective Module also had a small but significant effect on compassion when compared to retest. Thus, the ability to flexibly shift perspective from oneself to other persons and understand their intentions, beliefs and needs—as targeted by the Perspective Module—might constitute an additional "socio-cognitive route" to fostering compassion (as has been debated in the literature[5,31,32]). Training-related effects on compassion were smaller for the group practicing Affect without first learning how to stabilize the mind in the Presence Module. Thus, attention training during the Presence Module might prepare participants for the practices of the Affect Module.

Perspective was the only module to increase ToM performance beyond retest effects. This provides clear evidence that meditation-based mental training can increase performance in higher-order cognitive perspective taking, even in a healthy adult sample without any deficits in ToM. Two previous studies assessing inference of others' emotional states from eyes—a capacity

that is closely related but nevertheless dissociable from ToM[22]—yielded inconsistent results[23,24]. In contrast, the present results rely on a task validated for the specific assessment of high-level ToM performance [26,27], providing novel evidence for the malleability of this particular high-level cognitive perspective taking function. ToM deficits are associated to a range of clinical conditions, including autism, schizophrenia, and some forms of dementia[8], resulting in a high demand for effective interventions that might be informed by the finding of a highly specific training effect of the Perspective Module on social cognition. Given these effects were small in size, future research with longer training durations will be needed to explore whether then also large effect sizes can be achieved.

Finally, future research will need to explore the exact mechanisms of the different exercises within the training modules and disentangle their relative contributions to the observed changes in the three outcome measures. For example, both intersubjective training modules (Affect and Perspective) did not only contain classical meditation practices done by oneself (Loving-kindness and Observing Thoughts Meditation), but also so-called contemplative dyads practiced for 10 minutes with another partner as daily core practices supported by a web platform[33]. Thus, future investigations should ask to which extent such explicit intersubjective practices are needed to bring about lasting improvements in compassion and theory of mind.

In sum, the present results have two crucial implications: First, our findings clearly indicate that extended mental training effectively improves capacities that are crucial not only for individual flourishing, but also societal functioning at large. While executive control and attention are key predictors for educational success[34], compassion and ToM contribute to adaptive social functioning and communication, prosocial behavior, and economic decision making[30,35-37]. Second, the results clearly show that the type of practice really matters. Mindfulness practices

focused on present-moment awareness improve attention, but are not efficient in enhancing socio-affective and socio-cognitive skills. While the capacity to understand beliefs, desires, and needs of others, a crucial capacity in cross-cultural dialogues, can be improved through specific perspective taking training, socio-affective practices are best to foster a loving and compassionate attitude towards others. These findings are not only relevant for the increasing number of people who apply these techniques in their daily lives as a means for self-regulation and development[38]. Such differential mapping of mental training effects also has promising implications for the development of refined intervention programs in education, health, and labor settings as well as for clinical populations with deficits in the domains of attention, social affect, or social cognition.

Methods

Participants: Within two recruitment waves, a total of $N = 332$ healthy participants (197 female; mean age = 40.74, SD = 9.24; age range = 20-55) were selected for and agreed to participate in the study (see Singer et al., 2016[6], for a detailed description of the multi-step recruitment and screening procedure and characteristics of the final sample for each cohort). Since the study involves a large range of outcomes, the sample size was determined prior to recruitment based on practical considerations and previous studies in the field, which it exceeds in sample size[3]. From the first recruitment wave, 191 participants were selected and assigned to the RCC ($N = 30$), TC1 ($N = 80$), or TC2 ($N = 81$). From the second wave, 141 participants were selected and assigned to the RCC ($N = 60$) or to TC3 ($N = 81$). Assignment was done using a bootstrapping process which ensured that all cohorts were matched for age, gender, marital status, income, IQ, and a number of personality trait questionnaires (p for all comparisons > 0.1; see Singer et al., 2016[6]).

Across all four time points throughout the entire study, 78% (attention task) and 85% (EmpaToM) of the data were available and usable for analysis. As detailed in Table S1 in the Supplemental Material, missingness occurred due to study dropout/exclusion (6%), partial dropout/exclusion from MRI experiments (4%), technical, health, or scheduling issues at individual assessments (4% attention task, 5% EmpaToM), and poor or incorrect task performance (9%, only for the attention task). Criteria for poor or incorrect task performance were the same as in a previous study using baseline data from the attention task[25], that is, datasets with error-rates exceeding 50% in one of the experimental blocks or with a percentage of misses above 12.5% were excluded.

Finally, since our analysis focused on change scores (see below), the sample of the analysis was restricted to participants and time intervals where both pre- and post-scores were available (see Supplemental Material, Table S1 shows the number of datasets available for each time point, while Table S2 shows the number of change scores that could be calculated from these).

All participants gave informed consent prior to participation and the study was approved by the Research Ethics Committee of the University of Leipzig, number 376/12-ff and the Research Ethics Committee of the Humboldt University in Berlin, numbers 2013-02, 2013-29, and 2014-10. The study was registered with the Protocol Registration System of ClinicalTrials.gov under the title "Plasticity of the Compassionate Brain" with the ClinicalTrials.gov Identifier: NCT01833104.

Measures: Measures were assessed prior to training and during the last 5 weeks of each module. Assessments were done during a functional magnetic resonance imaging session (fMRI)—the respective imaging data will be reported in separate publications.

The cued flanker task assesses two main attention functions, executive control through flanker-

target conflict[39,40] and stimulus driven reorienting of attention through (invalid) spatial cueing of the target location[41,42], both in isolated and in concurrent demand conditions. Specifically, in each of 240 trials a cueing arrow indicated—with 80% valid and 20% invalid trials—whether the target appeared on the right or left side of the screen and participants indicated whether a target arrow—flanked by congruent or incongruent distracter arrows—pointed upwards or downwards (see figure 2a and Supplementary Methods for details). As previously reported on the baseline data of the present study[25], concurrent demand of stimulus-driven reorienting and executive control of attention (invalidly cued incongruent targets) causes over-additive increases in response costs, indicating that both functions rely on a common bottleneck or a general attentional capacity. To test for improvements in attention in the most comprehensive way and under the most challenging conditions, our analysis focused on the concurrent demand condition. For a more detailed picture, we also tested changes in the isolated markers of both functions.

The EmpaToM is a video task that has previously been validated for the assessment of core social cognitive and affective functions including compassion and ToM[26,27]. In each of 48 trials, the task presents a short video of a person describing a personal live event, which is either neutral or emotional, followed by ratings on affect and compassion, as well as either a factual reasoning (control) or a theory of mind question (see figure 2b and Supplementary Methods for a detailed task description). Compassion was assessed as the mean compassion rating across neutral and emotional videos. Since the conceptual understanding of compassion might change due to contemplative training (e.g., becoming aware of the difference between empathy and compassion), we ensured a consistent understanding by defining “compassion” during the EmpaToM training session as experiencing feelings of care, warmth, and benevolence towards another. .

ToM performance was assessed as mean accuracy in the ToM questions. Furthermore, in supplementary analyses we also report results for changes in RT as well as accuracy results controlled for changes in RT to rule out the possibility that accuracy improvements could be driven by a slowing of responses. Note that in previous cross-sectional studies on the EmpaToM task[26,27], for reduction of complexity, the main analysis was focused on an unweighted composite score of ToM performance because both measures, accuracy and RT, behaved alike, as shown in supplementary analyses of these studies. Here we focus on accuracy, as an improvement in accuracy due to the training would ultimately be more informative then changes in RT, under the condition that changes in speed-accuracy trade-off do not explain these improvements. However, for completeness we also report analyses with the composite score. Furthermore, we also assessed changes in factual reasoning questions to ensure that improvements would not be driven by general cognitive or motivational effects.

Statistical analysis: Data was analyzed using R software[43]. For each measure, we calculated mean scores per participant and time point. These scores were divided by the overall standard deviation to achieve comparability across measures. Change scores for each module and participant were then calculated by subtracting individual scores before each module from the scores at the end of each module and were entered into linear mixed model analysis. This approach relying on mixed effects modeling of change scores was chosen because it avoids biasing module change estimates by including different participants before and after a module, while allowing inclusion of participants who did not provide datasets at all time points. Furthermore, change scores can be modeled directly as a function of the different modules (or retest) and these can be contrasted against each other.

To evaluate hypotheses about specific effects of the three training modules on the targeted

measures, each module was contrasted against effects of other modules and of retest at the respective same time intervals. To this end, linear mixed models were estimated using the lme4 package[44]. Models included fixed effects for each time interval and module combination and random intercepts for participants. Specifically, for each outcome measure we fitted the following model to the change scores C_i:

$$C_i = \beta_0 + \beta_1 * retest_2 + \beta_2 * retest_3 + \beta_3 * Presence + \beta_4 * Affect_1 +$$
$$\beta_5 * Affect_2 + \beta_6 * Affect_3 + \beta_7 * Perspective_2 + \beta_8 * Perspective_3$$

Note that the first retest interval (i.e., $retest_1$) constitutes the intercept and all other effects are estimated in relation to this baseline. The fitted models then allowed us to test hypotheses of specific training effects by contrasting the respective parameter estimates against each other (see Supplementary Table S6 for a numeric specification of the contrasts).

Secondly, in order to test whether training module effects depended on time interval, we refitted the model with fixed effect factors for module (4 levels: retest, Presence, Affect, Perspective) and interval (3 levels: T0 to T1, T1 to T2, T2 to T3). Note that this model is equivalent to the first model in that it has the same amount of model parameters and the same model fit. Dependency of module effects on time interval (i.e., the interaction of module and interval) was then evaluated by comparing the full model against a model without the interaction term by means of chi-square likelihood ratio tests. A significant interaction would indicate that effects of Affect or Perspective might depend on the order in which they were completed (as first, second, or third module). In case of an interaction, we also report differential effects of the modules at each individual time interval.

Effect sizes for each module and time interval were calculated as suggested by Morris[45] for pretest-posttest-control group designs. Specifically, mean change in the retest participants was

subtracted from mean change in the training participants and divided by the pooled pretest standard deviation. Effects were classified according to standard conventions (i.e., small ≥ .20, medium ≥ .50, large ≥ .80).

To compare the effects that each module had on the three outcome measures, three separate mixed models were estimated for Presence, Affect, and Perspective Modules. The change scores pertaining to a given training module as well as retest scores from the same time intervals were entered into the model. Note that change scores were calculated from mean scores that had been divided by their standard deviation (see above), so that variance within each measure was equal. Models had fixed effects for intervention (one of the modules vs. retest) and outcome (attention, compassion, ToM) and random intercepts for participants. As retest effects might differ between the measures, the analysis focused on module by measure interactions, which indicate whether a module's effects contrasted against retest effects differed between the measures.

Following Bates and colleagues (2015)[44], assumptions of the fitted models were checked through visual inspection of residual plots (QQ-plots, residuals plotted against fitted values, scale-location plots), which did not indicate any violations of normality, linearity, and homoscedasticity. Throughout the manuscript, all *p*-values are based on two-tailed statistical tests. Currently, all the data acquired in the ReSource Project are saved in a local data base. Please send data requests to the corresponding author.

References

1 Goyal, M. *et al.* Meditation programs for psychological stress and well-being: a systematic review and meta-analysis. *JAMA Internal Medicine* **174**, 357-368, doi:10.1001/jamainternmed.2013.13018 (2014).

2 Sedlmeier, P. *et al.* The Psychological Effects of Meditation: A Meta-Analysis. *Psychological bulletin*, doi:10.1037/a0028168 (2012).

3 Tang, Y.-Y., Hölzel, B. K. & Posner, M. I. The neuroscience of mindfulness meditation. *Nature Reviews Neuroscience*, doi:10.1038/nrn3916 (2015).

4 Kabat-Zinn, J. *Full catastrophe living: using the wisdom of your body and mind to face stress, pain, and illness*. (Delacorte, 1990).

5 Dahl, C. J., Lutz, A. & Davidson, R. J. Reconstructing and deconstructing the self: cognitive mechanisms in meditation practice. *Trends in Cognitive Sciences* **19**, 515-523, doi:10.1016/j.tics.2015.07.001 (2015).

6 Singer, T. *et al. The ReSource Project. Background, design, samples, and measurements (2nd ed.)*. (Max Planck Institute for Human Cognitive and Brain Sciences, 2016).

7 Mitchell, J. P. Inferences about mental states. *Philosophical transactions of the Royal Society of London. Series B, Biological sciences* **364**, 1309-1316, doi:10.1098/rstb.2008.0318 (2009).

8 Brüne, M. & Brüne-Cohrs, U. Theory of mind—evolution, ontogeny, brain mechanisms and psychopathology. *Neuroscience and Biobehavioral Reviews* **30**, 437-455, doi:10.1016/j.neubiorev.2005.08.001 (2006).

9 Allen, M. *et al.* Cognitive-affective neural plasticity following active-controlled mindfulness intervention. *The Journal of neuroscience* **32**, 15601-15610, doi:10.1523/JNEUROSCI.2957-12.2012 (2012).

10 Slagter, H. A. *et al.* Mental training affects distribution of limited brain resources. *PLoS Biology* **5**, e138-e138, doi:10.1371/journal.pbio.0050138 (2007).

11 Tang, Y.-Y. *et al.* Short-term meditation training improves attention and self-regulation. *Proceedings of the National Academy of Sciences of the United States of America* **104**, 17152-17156, doi:10.1073/pnas.0707678104 (2007).

12 Salzberg, S. *Lovingkindness. The revolutionary art of happiness*. (Shambala, 1995).

13 Engen, H. G. & Singer, T. Compassion-based emotion regulation up-regulates experienced positive affect and associated neural networks. *Social Cognitive and Affective Neuroscience* **10**, 1291-1301, doi:10.1093/scan/nsv008 (2015).

14 Klimecki, O. M., Leiberg, S., Lamm, C. & Singer, T. Functional neural plasticity and associated changes in positive affect after compassion training. *Cerebral Cortex* **23**, 1552-1561, doi:10.1093/cercor/bhs142 (2013).

15 Klimecki, O. M., Leiberg, S., Ricard, M. & Singer, T. Differential pattern of functional brain plasticity after compassion and empathy training. *Social cognitive and affective neuroscience* **9**, 873-879, doi:10.1093/scan/nst060 (2014).

16 Kok, B. E. & Fredrickson, B. L. Upward spirals of the heart: Autonomic flexibility, as indexed by vagal tone, reciprocally and prospectively predicts positive emotions and social connectedness. *Biological psychology* **85**, 432-436, doi:10.1016/j.biopsycho.2010.09.005. (2010).

17 Brown, K. W. & Ryan, R. M. Perils and promise in defining and measuring mindfulness: Observations from experience. *Clinical Psychology: Science and Practice* **11**, 242-248, doi:10.1093/clipsy/bph078 (2004).

18 Grossman, P. On measuring mindfulness in psychosomatic and psychological research. *Journal of psychosomatic research* **64**, 405-408, doi:10.1016/j.jpsychores.2008.02.001 (2008).

19 Neff, K. D. & Germer, C. K. A pilot study and randomized controlled trial of the mindful self-compassion program. *Journal of Clinical Psychology* **69**, 28-44, doi:10.1002/jclp.21923 (2013).

20 Singer, T. The past, present and future of social neuroscience: a European perspective. *NeuroImage* **61**, 437-449, doi:10.1016/j.neuroimage.2012.01.109 (2012).

21 Lebois, L. a. M. *et al.* A shift in perspective: Decentering through mindful attention to imagined stressful Events. *Neuropsychologia* **75**, 505-524, doi:10.1016/j.neuropsychologia.2015.05.030 (2015).

22 Oakley, B. F. M., Brewer, R., Bird, G. & Catmur, C. 'Theory of Mind' is not Theory of Emotion: A cautionary note on the Reading the Mind in the Eyes Test. *Journal of Abnormal Psychology* **125**, 818-823 (2016).

23 Mascaro, J. S., Rilling, J. K., Negi, L. T. & Raison, C. L. Compassion meditation enhances empathic accuracy and related neural activity. *Social Cognitive and Affective Neuroscience* **8**, 48-55, doi:10.1093/scan/nss095 (2013).

24 Melloni, M. *et al.* Preliminary evidence about the effects of meditation on interoceptive sensitivity and social cognition. *Behavioral and Brain Functions* **9**, 1-6, doi:10.1186/1744-9081-9-47 (2013).

25 Trautwein, F.-M., Kanske, P. & Singer, T. Stimulus-driven reorienting impairs executive control of attention: Evidence for a common bottleneck in anterior insula. *Cerebral Cortex* (2016).

26 Kanske, P., Bockler, A., Trautwein, F.-M., Parianen Lesemann, F. H. & Singer, T. Are strong empathizers better mentalizers? Evidence for independence and interaction between the routes of social cognition. *Soc Cogn Affect Neurosci*, doi:10.1093/scan/nsw052 (2016).

27 Kanske, P., Böckler, A., Trautwein, F.-M. & Singer, T. Dissecting the social brain: Introducing the EmpaToM to reveal distinct neural networks and brain–behavior relations for empathy and Theory of Mind. *NeuroImage* **122**, 6-19, doi:10.1016/j.neuroimage.2015.07.082 (2015).

28 Jazaieri, H. *et al.* Enhancing compassion: a randomized controlled trial of a compassion cultivation training program. *Journal of Happiness Studies* **14**, 1113-1126, doi:10.1007/s10902-012-9373-z (2013).

29 Posner, M. I. *et al.* Attentional mechanisms of borderline personality disorder. *Proceedings of the National Academy of Sciences* **99**, 16366-16370 (2002).

30 Goetz, J. L., Keltner, D. & Simon-Thomas, E. Compassion: an evolutionary analysis and empirical review. *Psychological bulletin* **136**, 351-374, doi:10.1037/a0018807 (2010).

31 Engen, H. G. & Singer, T. Affect and motivation are critical in constructive meditation. *Trends in Cognitive Sciences* **20**, 159-160, doi:10.1016/j.tics.2015.11.004 (2015).

32 Dahl, C. J., Lutz, A. & Davidson, R. J. Cognitive processes are central in compassion meditation. *Trends in Cognitive Sciences* **20**, 161-162, doi:10.1016/j.tics.2015.12.005 (2016).

33 Kok, B. E. & Singer, T. Contemplating the other - Introducing the Contemplative Dyad and its effects on engagement, social closeness, and self-disclosure over six months of mental training via a randomized clinical trial. *JAMA Psychiatry* (under review).

34 Checa, P. & Rueda, M. R. Behavioral and brain measures of executive attention and school competence in late childhood. *Developmental Neuropsychology* **36**, 1018-1032, doi:10.1080/87565641.2011.591857 (2011).

35 Morishima, Y., Schunk, D., Bruhin, A., Ruff, C. C. & Fehr, E. Linking brain structure and activation in temporoparietal junction to explain the neurobiology of human altruism. *Neuron* **75**, 73-79, doi:10.1016/j.neuron.2012.05.021 (2012).

36 Weng, H. Y., Fox, A. S., Hessenthaler, H. C., Stodola, D. E. & Davidson, R. J. The Role of Compassion in Altruistic Helping and Punishment Behavior. *Plos One* **10**, e0143794-e0143794, doi:10.1371/journal.pone.0143794 (2015).

37 Hein, G., Morishima, Y., Leiberg, S., Sul, S. & Fehr, E. The brains functional network architecture reveals human motives. *Science* **351**, 1074-1078, doi:10.1126/science.aac7992 (2016).

38 Barnes, P. M. & Bloom, B. Complementary and Alternative Medicine Use Among Adults and Children: United States, 2007. *National Health Statistics Reports: USDHHS* **12** (2008).

39 Eriksen, B. A. & Eriksen, C. W. Effects of noise letters upon the identification of a target letter in a nonsearch task. *Perception & Psychophysics* **16**, 143-149 (1974).

40 Fan, J., McCandliss, B. D., Sommer, T., Raz, A. & Posner, M. I. Testing the efficiency and independence of attentional networks. *Journal of Cognitive Neuroscience* **14**, 340-347, doi:10.1162/089892902317361886 (2002).

41 Posner, M. I. Orienting of attention. *Quarterly Journal of Experimental Psychology* **32**, 3-25 (1980).

42 Corbetta, M., Kincade, J. M., Ollinger, J. M., McAvoy, M. P. & Shulman, G. L. Voluntary orienting is dissociated from target detection in human posterior parietal cortex. *Nature Neuroscience* **3**, 292-297, doi:10.1038/73009 (2000).

43 R: a language and environment for statistical computing (R Foundation for Statistical Computing, Vienna, Austria, 2013).

44 Bates, D., Mächler, M., Bolker, B. & Walker, S. Fitting linear mixed-effects models using lme4. *Journal of Statistical Software* **67**, doi:10.18637/jss.v067.i01 (2015).

45 Morris, S. B. Estimating effect sizes from pretest-posttest-control group designs. *Organizational Research Methods* **11**, 364-386, doi:10.1177/1094428106291059 (2008).

46 Petersen, S. E. & Posner, M. I. The attention system of the human brain: 20 years after. *Annual Review of Neuroscience* **35**, 73-89, doi:10.1146/annurev-neuro-062111-150525 (2012).

Acknowledgments:

Tania Singer, as principal investigator, received funding for the *ReSource Project* from a) the European Research Council under the European Community's Seventh Framework Program (FP7/2007-2013/ ERC Grant Agreement Number 205557 to T.S.), and b) from the Max Planck Society. We are thankful to the members of the Social Neuroscience Department involved in the *ReSource Project* over many years, in particular to the teachers of the *ReSource* intervention program, to Astrid Ackermann, Christina Bochow, Matthias Bolz and Sandra Zurborg for managing the large-scale longitudinal study, to Elisabeth Murzik, Sylvia Tydecks , Kerstin Träger, and Nadine Otto for help with recruiting and data archiving, to Henrik Grunert for technical assistance, to Manuela Hofmann, Sylvie Neubert, and Nicole Pampus for help with data collection, and to Hannes Niederhausen and Torsten Kästner for data management. Thank you also to the research assistants, especially to Theo Schäfer for help with data analysis.

Author Contributions:

T.S. initiated and developed the ReSource Project and model as well as the training protocol and secured all funding. All authors contributed to the present study design and development of the tasks. F.-M. Trautwein and P. Kanske were involved in testing and data collection. F.-M. Trautwein performed the data analysis and interpretation under the supervision of T. Singer, P. Kanske and A. Böckler. All authors contributed to writing up or revising the paper and approved the final version of the manuscript for submission.

Competing interests: The authors declare no competing financial interest.

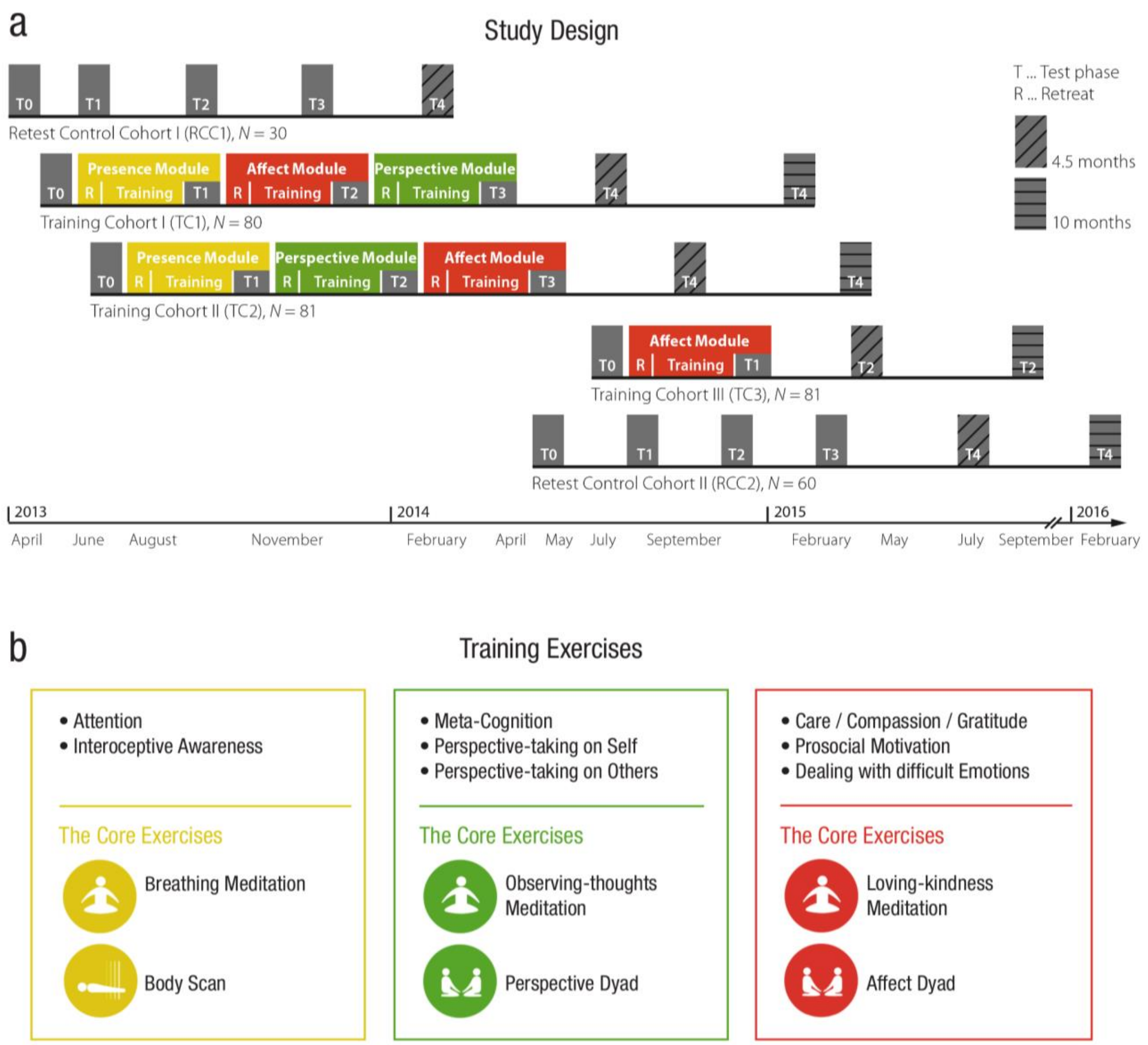

Fig. 1. Study design. (a) Timeline of training (colored areas) and data collection (gray areas) for the training and retest control cohorts. The modules were completed in different orders, allowing using these as active control conditions for each other. For practical testing reasons, all cohorts proceeded in a shifted manner, and retest participants were split into two cohorts but are jointly analyzed. Retest cohorts completed the measurements without any training. The full *ReSource* Design as shown in the figure also included follow-up assessments; however, these are not included in the present investigation. (b) Illustration of core exercises of the three modules: Presence (yellow), Affect (red), Perspective (green). Please refer to the Supplementary Material for details. Figures were adapted from Singer et al., (2016).

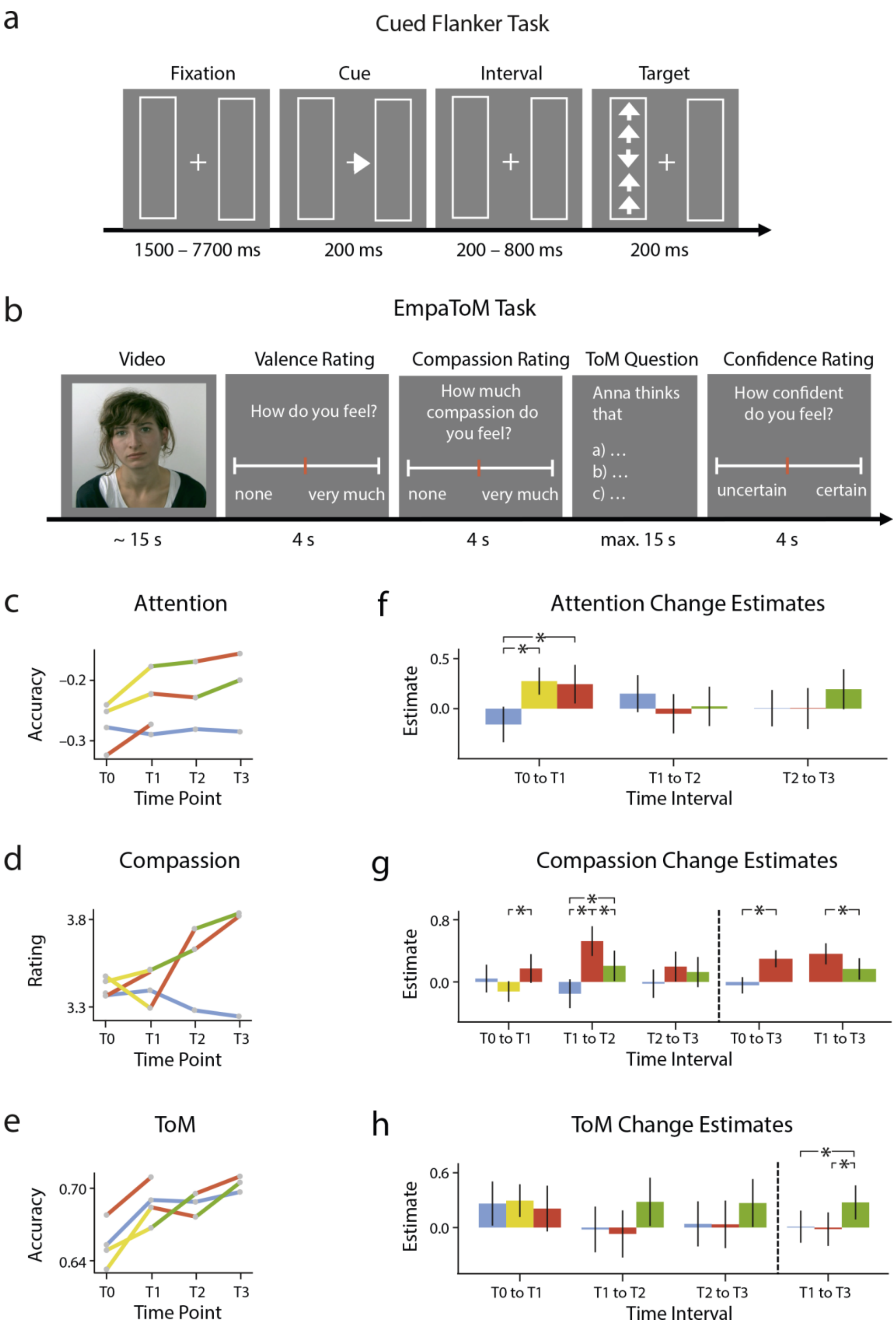
a
Cued Flanker Task
Fixation
Cue
Interval
Target
1500 – 7700 ms
200 ms
200 – 800 ms
200 ms
b
EmpaToM Task
Video
Valence Rating
Compassion Rating
ToM Question
Confidence Rating
How do you feel?
none
very much
How much compassion do you feel?
none
very much
Anna thinks that
a) …
b) …
c) …
How confident do you feel?
uncertain
certain
~ 15 s
4 s
4 s
max. 15 s
4 s
c
Attention
Accuracy
−0.2
−0.3
T0
T1
T2
T3
Time Point
f
Attention Change Estimates
Estimate
0.5
0.0
T0 to T1
T1 to T2
T2 to T3
Time Interval
d
Compassion
Rating
3.8
3.3
T0
T1
T2
T3
Time Point
g
Compassion Change Estimates
Estimate
0.8
0.0
T0 to T1
T1 to T2
T2 to T3
T0 to T3
T1 to T3
Time Interval
e
ToM
Accuracy
0.70
0.64
T0
T1
T2
T3
Time Point
h
ToM Change Estimates
Estimate
0.6
0.0
T0 to T1
T1 to T2
T2 to T3
T1 to T3
Time Interval

Fig. 2. Illustration of behavioral tasks, descriptive data, and model estimates. (a) Illustration of one trial of the cued flanker task that was used to assess attention. The task combines spatial cueing, inducing reorienting of attention through invalidly cued targets[41,42], with flanker-target conflict, requiring executive control of attention in incongruent trials[39,40], and thus allows assessing shared and isolated resources of these hallmarks of attention[25,46]. (b) Illustration of one trial of the EmpaToM task that was used to assess trial-wise experience of compassion and accuracy of mental state inference (i.e. ToM). The task has previously been validated to independently assess these socio-affective and socio-cognitive capacities[26,27]. Note that this simplified illustration omits fixation periods between the screens and the name of the speaker presented in the beginning of each trial. For details of the tasks, please refer to the methods section. (c-e) Descriptive plots of mean values for attention (difference of correct response proportions in the reorienting and conflict condition minus baseline condition), compassion (mean ratings on a scale from 0 to 6) and ToM (proportion of correct responses in ToM questions) per time point and group. Note that differences between groups at T0 were not significant (*F*-Test *p*-values all > .11). The mean values of each individual were used to calculate change scores for each available pair of two consecutive time points, which were used to estimate effects for retest and the three training modules shown in (f-h). In panels (g) and (h), to the right of the dashed lines, estimates are averaged across time intervals as was done to test the main hypotheses for these two measures. Error bars in (f-h) indicate 95% confidence intervals.

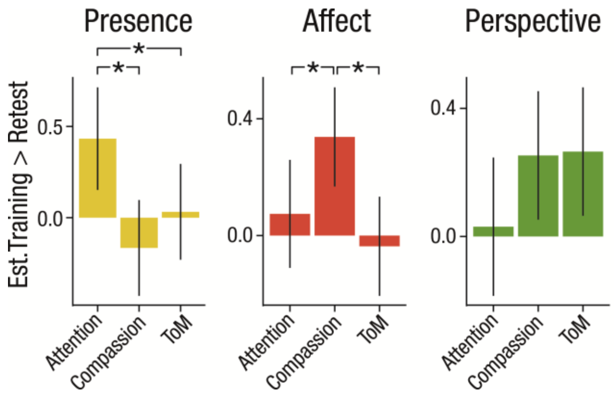

Fig. 3. Contrasts of training vs. retest effects estimated from standardized change scores of attention, compassion and ToM. Estimates from three different models are shown assessing effects of Presence (T0 to T1 scores), Affect (T0 to T1, T1 to T2, and T2 to T3 scores), and Perspective (T1 to T2 and T2 to T3 scores). Error bars indicate 95% confidence intervals.
